# Seasonality and trend prediction of scarlet fever incidence in mainland China from 2004 to 2018 using a hybrid SARIMA-NARX model

**DOI:** 10.7717/peerj.6165

**Published:** 2019-01-17

**Authors:** Yongbin Wang, Chunjie Xu, Zhende Wang, Juxiang Yuan

**Affiliations:** 1 School of Public Health, North China University of Science and Technology, Tangshan, China; 2 School of Public Health, Capital Medical University, Beijing, China

**Keywords:** SARIMA model, Scarlet fever, NAR model, Hybrid model, Forecasting, NARX model, Incidence cases, Seasonality, Trend, In mainland China

## Abstract

**Background:**

Scarlet fever is recognized as being a major public health issue owing to its increase in notifications in mainland China, and an advanced response based on forecasting techniques is being adopted to tackle this. Here, we construct a new hybrid method incorporating seasonal autoregressive integrated moving average (SARIMA) with a nonlinear autoregressive with external input(NARX) to analyze its seasonality and trend in order to efficiently prevent and control this re-emerging disease.

**Methods:**

Four statistical models, including a basic SARIMA, basic nonlinear autoregressive (NAR) method, traditional SARIMA-NAR and new SARIMA-NARX hybrid approaches, were developed based on scarlet fever incidence data between January 2004 and July 2018 to evaluate its temporal patterns, and their mimic and predictive capacities were compared to discover the optimal using the mean absolute percentage error, root mean square error, mean error rate, and root mean square percentage error.

**Results:**

The four preferred models identified were comprised of the SARIMA(0,1,0)(0,1,1)_12_, NAR with 14 hidden neurons and five delays, SARIMA-NAR with 33 hidden neurons and five delays, and SARIMA-NARX with 16 hidden neurons and 4 delays. Among which presenting the lowest values of the aforementioned indices in both simulation and prediction horizons is the SARIMA-NARX method. Analyses from the data suggested that scarlet fever was a seasonal disease with predominant peaks of summer and winter and a substantial rising trend in the scarlet fever notifications was observed with an acceleration of 9.641% annually, particularly since 2011 with 12.869%, and moreover such a trend will be projected to continue in the coming year.

**Conclusions:**

The SARIMA-NARX technique has the promising ability to better consider both linearity and non-linearity behind scarlet fever data than the others, which significantly facilitates its prevention and intervention of scarlet fever. Besides, under current trend of ongoing resurgence, specific strategies and countermeasures should be formulated to target scarlet fever.

## Introduction

Scarlet fever is an acute respiratory contagious disease as a consequence of *group A streptococcus pyogenes* (GAS) infection (You et al., 2018). The bacteria can frequently be spread by coughing or sneezing of the patients or carriers (Zhang et al., 2017), among whom children are fairly susceptible to the infections, particularly in the age of 5 to 15 years (Zhang et al., 2017). The clinical signs and symptoms of the infected are commonly characterized by a fever, angina, diffuse red rash of the whole body and an obvious desquamation after rash (Zhang et al., 2017), while a small number of patients can also develop heart, kidney and joint damage due to allergies after illness (Luk et al., 2012). The disease was among the major causes for serious illnesses in children in the early 20th century across the world (Lamagni et al., 2018), and since then this life-threatening illness has been well controlled as a result of the scale-up of antibiotics, together with the improvement of living standards (Lamagni et al., 2018). However, over the past decade, an exceptional upside in the morbidity of scarlet fever has occurred in some Asian and European countries and areas, containing mainland China (Liu et al., 2018), Vietnam (Andrey & Posfay-Barbe, 2016), Hong Kong (Luk et al., 2012), South Korea (Kim & Cheong, 2018), Australia (Feeney et al., 2005), Germany (Brockmann, Eichner & Eichner, 2018) and England (Lamagni et al., 2018). This worsening trend is becoming increasingly fierce, especially in China where the ongoing resurgence in disease morbidity has exerted a marked influence on Chinese population since 2011 and there still is a current scarcity of an available vaccine against scarlet fever (Lamagni et al., 2018; Liu et al., 2018; Walker & Brouwer, 2018; Wong & Yuen, 2018; Zhang et al., 2016a; Zhang et al., 2016b; Zhang & Liu, 2018). Consequently, faced with such a serious public health issue, to better provide an unambiguous and quantitative direction for the future resource utilization and development of prevention and control plans of this disease, a reliable forecasting approach with robust accuracy and precision to detect the epidemic patterns of scarlet fever in the near future is required.

At present, many efforts have been made to construct modeling approaches to track and understand the temporal characteristics of infectious diseases, and furthermore to predict outbreaks (He et al., 2017). A multitude of standard mathematical techniques like the autoregressive integrated moving average (ARIMA) model (Song et al., 2016), support vector machine (Liang et al., 2018), multivariate time series method (Zhang et al., 2016a), generalized regression model (Zhang et al., 2016b), error-trend-seasonal technique (Wang et al., 2018), seasonal decomposition model and exponential smoothing model (Al-Sakkaf & Jones, 2014), have been regarded as a serviceable policy-supportive tool for the incidence time series forecasting of contagious diseases. Of these approaches, the ARIMA method assuming time series to be stationary is the most popular approach for time series estimation. Generally, the morbidity data of infectious diseases are commonly affected and constrained by the time-varying trends, cyclicity, seasonal variation and random fluctuation (He et al., 2017). These facets make the data show complex linear and nonlinear interactions. However, the ARIMA method that essentially belongs to a linear model has a limited capacity to unearth the non-stationarity and non-linearity behind the data (Zhou et al., 2018). In order to capture the uncertainty in the data, artificial neural networks (ANNs) have attracted much attention in the past years as they have been attested to exhibit a powerful nonlinear mapping ability (Zhou et al., 2018). Hence, recent years have seen increasingly rapid advances in the field of epidemiological predictions using hybrid methods combining the linear and nonlinear models (He et al., 2017; Wei et al., 2017; Wu et al., 2015; Zhou et al., 2018). Among the combined methods favoring better development in the forecasting accuracy for time series relative to other combinations, single ARIMA or ANNs models employed solely is such a hybrid technique integrating the ARIMA with a nonlinear auto-regressive neural network (NAR) (Wang et al., 2017; Wu et al., 2015; Yu et al., 2014; Zhou et al., 2014a). Yet recent finding demonstrated the hybrid ARIMA-NAR technique failed to be as good as the separate use of the NAR model for predicting the number of new admission inpatients (Zhou et al., 2018). Thus, the ARIMA–NAR method is invariably not beneficial for forecasting the diseases time series, and this traditional combined approach may be meliorated in some contexts.

It is well known that time variable can offer significantly useful information in the incidence forecasting of infectious diseases including notable seasonality and periodicity (Wu et al., 2015). However, this component is commonly ignored in fitting a time series. Furthermore, as highlighted by many researches, scarlet fever is an illness with evident seasonal characteristics (Kim & Cheong, 2018; Lamagni et al., 2018; Liu et al., 2018; Zhang et al., 2016b). As far as we are aware, the time variable has not been considered in an ARIMA-NAR model with regard to modeling the incidence cases of scarlet fever before. Therefore, inspired by this pattern, we aimed to establish a seasonal ARIMA (SARIMA) model, a NAR model, a traditional SARIMA-NAR approach, and a novel SARIMA-NAR with external input approach, specified as SARIMA-NARX, and then these four methods were employed to simulate and estimate the scarlet fever morbidity data in mainland China intended to seek a preferred technique for detecting and warning its temporal trends in advance. We expect that the approach will indeed be valuable in the prevention and control of scarlet fever.

## Materials & Methods

### Data collection

In this study, the monthly notified cases of scarlet fever from January 2004 to July 2018 came from the notifiable infectious disease monitoring system provided by the Chinese Center for Disease Control and Prevention(CDC) (http://www.nhfpc.gov.cn/jkj/s3578%20/new_list.shtml), and the annualized population data between 2004 and 2017 were retrieved from National Bureau of Statistics of China (http://data.stats.gov.cn/easyquery.htm?cn=C01) (File S1). A total of 175 months’ observations spanning 15 years were aggregated as the analytical data. Afterwards, to evaluate and validate the performance of these four approaches used, we selected the observations from January 2004 to December 2017 as the in-sample training horizons (168 points), whereas the rest data from January 2018 to July 2018 were utilized for the out-of-sample verification horizons (also see Table S1).

Based on the 2004 Chinese Contagious Diseases Law, the cases identified by the clinicians or laboratory-confirmed diagnosis must be reported to the above-mentioned monitoring system within 24 h and the duplicate cases must be smoothed away by the professionals at the end of the same month. Since the reported cases of scarlet fever were assembled as a secondary data absent from detailed individual information, the ethical approval or consent failed thus to be needed.

### Establishment of the basic SARIMA model

As depicted above, the scarlet fever incidence series showed obvious cyclicality and seasonality over time, a classical SARIMA method, designated as SARIMA(p, d, q) (P, D, Q)_s_, should be considered to erect the benchmark model. In the process of forming this model, the seasonality of scarlet fever was treated as the explanatory variable and monthly scarlet fever as the response variable, and its defining equation can be written as

(1) \begin{eqnarray*} \left\{ \begin{array}{@{}l@{}} \displaystyle \varphi \left( B \right) \Phi \left( {B}^{\mathrm{s}} \right) {\Delta }^{d}{\Delta }_{s}^{D}{X}_{t}=\theta \left( B \right) \Theta \left( {B}^{s} \right) {\varepsilon }_{t}\\ \displaystyle E \left( {\varepsilon }_{t} \right) =0, Var \left( {\varepsilon }_{t} \right) ={\sigma }_{\varepsilon }^{2},E \left( {\varepsilon }_{t}{\varepsilon }_{s} \right) =0,s\not = t\\ \displaystyle E \left( {X}_{s}{\varepsilon }_{t} \right) =0,{\forall }_{s}\lt t \end{array} \right. \end{eqnarray*}

where, B is the backward shift operator, ε_*t*_ denotes the residuals from scarlet fever data, S is the periodicity of scarlet fever incidence series, d and D are the non-seasonal and seasonal differenced times, respectively. p and q are the orders of autoregressive model and moving average model, respectively. P and Q are the orders of seasonal autoregressive model and moving average model, respectively. ${\nabla }^{d}={ \left( 1-B \right) }^{d}$, ${\mathop{\nabla }\nolimits }_{S}^{D}={ \left( 1-B \right) }^{SD}$, $\phi \left( B \right) =1-{\phi }_{1}B-\cdots -{\phi }_{p}{B}^{p},\theta \left( B \right) =1-{\theta }_{1}B-\cdots -{\theta }_{q}{B}^{q}$, $\Phi \left( {B}^{s} \right) =1-{\Phi }_{1}{B}^{s}-\cdot \cdot \cdot -{\Phi }_{P}{B}^{Ps}$, $\Theta \left( {B}^{s} \right) =1-{\Theta }_{1}{B}^{s}-\cdots -{\Theta }_{Q}{B}^{Qs}$.

In SPSS software, the key parameters (p, d, q, P, D and Q) for the optimal method included in all candidate models could automatically be identified by performing the “Expert Modeler” function based on either the largest value of the coefficient of determination (*R*^2^) or the lowest value of the normalized schwarz bayesian criterion (SBC). Subsequently, the mimic and predictive results were given by the selected best-fitting method. Ultimately, the autocorrelation function (ACF) and partial autocorrelation function (PACF) plots of the residuals, and Ljung–Box *Q* test were adopted to diagnose whether the estimated residuals met the demand of a white-noise series (Al-Sakkaf & Jones, 2014; Song et al., 2016; Wu et al., 2015).

### Construction of the basic NAR model

In the real-world scenario, the uncertainty and complex nonlinear traits hidden behind the infectious incidence are not easily excavated by the linear models (Wu et al., 2015). At this time, ANNs will be of great help in unveiling the complexities of this phenomenon because they are capable of approximating arbitrarily intricate irregular series to attain any desired accuracy by dint of their powerful flexible nonlinear mapping capability (Wei et al., 2017; Wu et al., 2015; Zhou et al., 2014a). Currently, among the ANNs having an outstanding forecasting ability is the NAR technique that is one of dynamic recurrent neural networks with embedded memory, and has emerged as a powerful tool in estimating dynamical systems and studying the behaviors of highly non-stationary and nonlinear series(He et al., 2017; Zhou et al., 2014a). The architecture of the basic NAR method is illustrated as Fig. S1, and its formula can be written as:

(2) \begin{eqnarray*}y(t)=f(y(t-1),y(t-2),\ldots ,y(t-d))\end{eqnarray*}

where *y*(*t*) refers to the forecasting points of scarlet fever incidence series only depended on the prior data of lagged period *d*.

In order to find the best-simulating NAR model. Initially, the whole observed data used to train the network were randomly allocated into three parts including training with 80% of the observations, validation with 10% and testing with 10%. Among which, the training dataset played a significant role in determining the network parameters; the validation dataset was utilized to improve the model’s generalization by avoiding overfitting; the testing dataset provided an independent measure of the model performance (Zhou et al., 2014a). Subsequently, we repeatedly adjust the number of hidden neurons and delays *d* to seek the preferred model in an open feedback loop according to the residual ACF plot and response plot of outputs and targets, along with the mean square error(MSE) and correlation coefficient (*R*) (Wu et al., 2015). Finally, the open-loop mode derived should be transformed to closed-loop form for multistep-ahead predictions (Wu et al., 2015).

### Erection of the SARIMA-NAR hybrid model

As illustrated above, mining the linear component in the incidence series of scarlet fever is what the SARIMA approach specializes in, whereas the residual errors constitute the nonlinear element that this model is unable to analyze. Fortunately, the NAR technique thought of as a function approximator can provide a deeper insight into analysis for this component (Zhou et al., 2018). Driven by the merit of NAR method, a hybrid SARIMA-NAR technique was thus built to develop a deeper understanding of the epidemic trends in scarlet fever morbidity owing to its comprehensive consideration for their own characteristics and complementary advantages of these two basic models. In such a combined method, the residual error series generated by the SARIMA approach was used to build a basic NAR model. Next, the dataset groupings, modeling procedures and performance assessment during construction of the combined model were conducted as such in the basic NAR method. Finally, the results mimicked and forecasted by the SARIMA and NAR models employed separately were summed to become the ultimate scarlet fever morbidity cases derided from the combined methods. The architecture of this traditional hybrid model is presented as Fig. S2, and the specified equation is described by:

(3) \begin{eqnarray*}& & \hat {e}(t)=f(e(t-1),e(t-2),\ldots ,e(t-d))\end{eqnarray*}

(4) \begin{eqnarray*}& & \hat {y}={\hat {a}}_{t}+{\hat {e}}_{t}\end{eqnarray*}

where $\hat {y}$ is the fitted and forecasted incidence cases with this hybrid method, ${\hat {a}}_{t}$ denotes the simulations and predictions of SARIMA model, ${\hat {e}}_{t}$ represents the values derived from the fitted and predicted relied merely on the SARIMA residual series of lagged period *d*.

### Development of the SARIMA-NARX hybrid model

Seasonal changes have proved to be particularly valuable to the occurrence and control of infectious diseases and also vital to forecast trends (Yang et al., 2017). As presented in the basic NAR or traditional SARIMA-NAR approaches, these two techniques all adopted the known historical data irrespective of other drivers to forecast the future unknown data. During training these models, the time variable is invariably neglected, which may not be conducive to the development in forecasting performance particularly for infectious diseases with manifest seasonality and periodicity. In general, the nonlinear information was contained in the residuals yielded by the SARIMA model (Zhou et al., 2014a), provided that the association between the predictive results from SARIMA method and the observed values can be evaluated, the remaining clues of the data will be extracted. Consequently, in the SARIMA-NARX approach, the time variable and values mimicked and forecasted by the SARIMA method were viewed as the input variables and the actual data as the values to be predicted, and then both the linear and nonlinear components were captured. Subsequently, the dataset divisions, modeling steps and performance evaluation during development of the hybrid approach were identical to the basic NAR method. The architecture of this proposed hybrid approach is depicted as Fig. S3, and its equation is:

(5) \begin{eqnarray*}\hat {y}(t)=f(y(t-1),\ldots ,y(t-d),x(t-1),\ldots ,x(t-d))\end{eqnarray*}

where, $\hat {y}$ is the mimic and forecasted incidence with this hybrid technique, *y* is the given prior scarlet fever incidence data of lagged period *d*. *x* stands for the inputs including the time variable as well as the stimulations and forecasts from the SARIMA approach.

### Model performance evaluation

Four performance indices were computed in the in-sample simulating errors and out-of-sample forecasting errors to judge the accuracy of models. Selection for the preferred model could be done by the mean absolute percentage error (MAPE), root mean square error (RMSE), mean error rate (MER), and root mean square percentage error (RMSPE); the model with the smallest values of these indices should be identified as the optimal.

(6) \begin{eqnarray*}& & \text{RMSE}=\sqrt{ \frac{1}{N} \sum _{i=1}^{N}({X}_{i}-{\hat {X}}_{i})^{2}}\end{eqnarray*}

(7) \begin{eqnarray*}& & \text{MAPE}= \frac{1}{N} \sum _{i=1}^{N} \frac{ \left\vert {X}_{i}-{\hat {X}}_{i} \right\vert }{{X}_{i}} \end{eqnarray*}

(8) \begin{eqnarray*}& & \text{MER}= \frac{ \frac{1}{N} \sum _{i=1}^{N} \left\vert {X}_{i}-{\hat {X}}_{i} \right\vert }{{\overline{X}}_{i}} \end{eqnarray*}

(9) \begin{eqnarray*}& & \text{RMSPE}=\sqrt{ \frac{1}{N} \sum _{i=1}^{N}( \frac{{X}_{i}-{\hat {X}}_{i}}{{X}_{i}} )^{2}}\end{eqnarray*}

Here, *X*_*i*_ denotes the actual observations, ${\hat {X}}_{i}$ represents the simulated and forecasted values with the chosen methods, ${\overline{X}}_{i}$ is the mean of the actual observations, *N* refers to the number of mimics and forecasts.

### Statistical process

The SARIMA method was developed with SPSS software (version 17.0, IBM Corp, Armonk, NY), the NAR, SARIMA-NAR, and SARIMA-NARX models were formed using MATLAB software (version R2014a; MathWorks, Natick, MA, USA). Meanwhile, to examine whether there exists conditional heteroskedastic behaviour and volatility (ARCH effect) in the errors produced by these methods, the Lagrangian multiplier (LM) test was undertaken in the residuals from all models. A *P* value <0.05 was considered significant.

## Results

### General information

**Figure 1:**
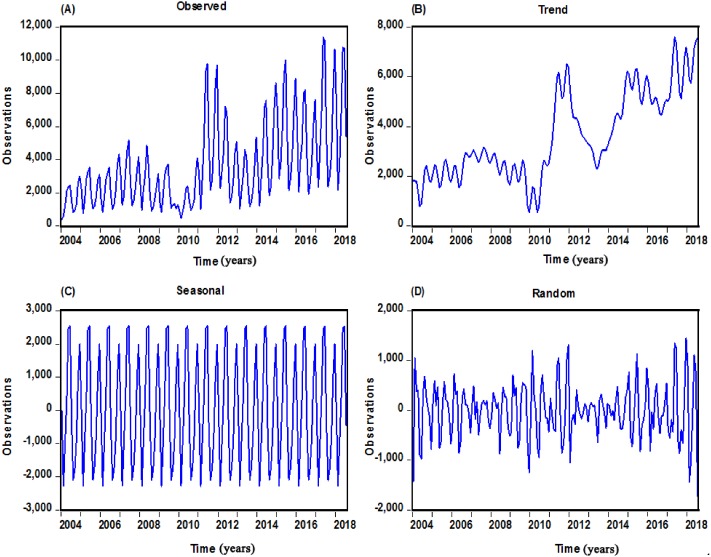
Monthly scarlet fever cases notified from January 2004 to July 2018 in mainland China and decomposed trend, seasonal and random components with the additive seasonal decomposition. (A) The actual scarlet fever cases notified from January 2004 to July 2018; (B) The decomposed trend trait of scarlet fever; (C) The decomposed seasonal trait of scarlet fever; (D) The decomposed random fluctuation trait of scarlet fever.

Over the period of January 2004 to July 2018, a substantial rising trend (on average, 9.641% annually) of scarlet fever case notifications was observed, the total cases of 630,031 were notified with an average monthly cases of 3,601, leading to an average annual incidence rate of 3.063 per 100,000 people. According to the 15 whole years of data, the maximum number of case notifications in 2017 have reached 74,369(5.350 per 100,000 persons), which is almost four-fold than that of 2004 when it was only 18,939(1.457 per 100,000 population) in all with the lowest level (Fig. S4). When the additive seasonal decomposition was employed to analyze the secular change and cyclicity, the case numbers retained relatively low and steady through 2004 to 2010 (total 175,841 cases) with an acceleration of 1.10% annually, while a sudden escalation was noted in 2011 with 63,878 cases (4.741 per 100,000 people), and then continued to upsurge for the remaining period (on average, 12.689% annually), apart from the year of 2013 (Fig. 1 and S5). Besides, scarlet fever could occur throughout the year, yet case notifications had a distinct seasonal distribution across China and showed double peak pattern in all years, there were few cases in February, a sharp increase in cases between March and June, high levels between May and June, with a decline in cases through July to October, but with a secondary peak during November and December of these years (Fig. S6). The summer peak appears to have gotten larger over the time series.

### The best-performing SARIMA model

**Figure 2:**
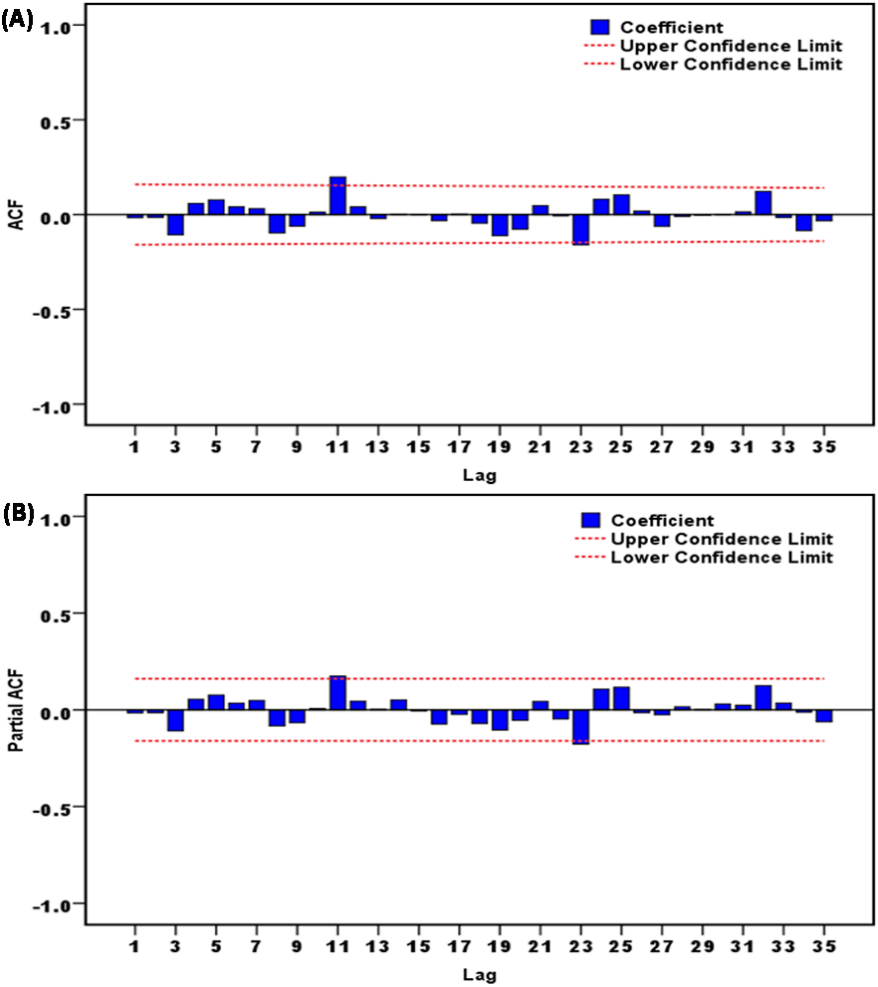
Correlation function graphs of residuals from SARIMA(0,1,0)(0,1,1)_12_ model for scarlet fever morbidity time series. (A) Autocorrelation function (ACF) graph of residuals; (B) partial autocorrelation function (PACF) graph of residuals. As illustrated in this graph, no correlation coefficients were observed beyond the 95% uncertainty bounds except for these points at 11 and 23 lags, which is also reasonable because the higher-order correlation may occasionally exceed the limits. These results intimated that the chosen SARIMA model was appropriate.

In the SARIMA construction, by performing the time series modeler in the designated in-sample data, the software automatically chose the SARIMA(0,1,0)(0,1,1)_12_ as the best-fitting specification, the fit statistics were followed by the largest *R*^2^ of 0.938 and the lowest normalized BIC of 12.864. Diagnostic checking for the fitness of the SARIMA method displayed the key parameter obtained was statistically significant with SMA = 0.795 (*t* = 10.597, *P* < 0.001), and based on its autocorrelation analysis of errors (Fig. 2), along with the Ljung–Box Q and LM tests of errors (Tables 1 and 2), it can be seen that all the *P*-values were greater than 0.05, revealing the errors were in close proximity to actualize a complete white noise sequence and no remaining ARCH effect was found in this residual error series. According to these results from the errors, we confirmed that this identified preferred SARIMA method was suited to implement forecasting for the out-of-sample data. The equation of the SARIMA (0, 1, 0) (0, 1, 1)_12_ approach can be defined as $ \left( 1-B \right) \left( 1-{B}^{12} \right) {X}_{t}= \left( 1-0.795{B}^{12} \right) {\varepsilon }_{t}$.

**Table 1:** Ljung-Box *Q* tests of the residuals for the identified four optimal models.

**Lags**	**SARIMA**		**NAR**		**SARIMA-NAR**		**SARIMA-NARX**	
	**Box-Ljung Q**	***P***	**Box-Ljung Q**	***P***	**Box-Ljung Q**	***P***	**Box-Ljung Q**	***P***
1	0.044	0.834	0.175	0.675	0.281	0.596	0.427	0.514
3	1.937	0.586	2.196	0.533	3.098	0.377	2.188	0.534
6	3.703	0.717	3.047	0.803	5.567	0.473	3.572	0.734
9	6.068	0.733	6.273	0.712	5.832	0.757	4.080	0.906
12	12.994	0.369	8.367	0.756	7.642	0.812	4.556	0.971
15	13.066	0.597	12.891	0.611	8.396	0.907	5.320	0.989
18	13.626	0.753	15.683	0.615	12.460	0.823	6.156	0.996
21	17.284	0.694	16.792	0.724	12.716	0.918	9.658	0.983
24	23.193	0.508	19.568	0.721	26.301	0.338	14.877	0.924
27	26.000	0.519	20.085	0.827	33.785	0.172	17.910	0.906
30	26.024	0.674	21.977	0.855	34.576	0.258	18.405	0.952
33	29.048	0.664	26.962	0.761	39.324	0.208	21.217	0.944
36	41.331	0.249	31.613	0.677	41.618	0.239	22.243	0.965

**Notes.**

SARIMA seasonal autoregressive integrated moving average model NAR nonlinear auto-regressive neural network model NARX nonlinear auto-regressive with external input neural network

**Table 2:** LM tests of the actual observations and residuals for the identified four optimal models.

**Lags**	**Observed**		**SARIMA**		**NAR**		**SARIMA-NAR**		**SARIMA-NARX**	
	**LM-test**	***P***	**LM-test**	***P***	**LM-test**	***P***	**LM-test**	***P***	**LM-test**	***P***
1	68.565^*^	<0.001	0.000	0.984	0.187	0.665	0.139	0.709	0.935	0.334
3	99.672^*^	<0.001	6.806	0.078	1.541	0.673	0.439	0.932	1.355	0.716
6	126.860^*^	<0.001	8.658	0.194	2.550	0.863	1.230	0.975	2.618	0.855
9	125.480^*^	<0.001	11.234	0.260	7.034	0.634	1.431	0.998	3.401	0.946
12	125.820^*^	<0.001	12.253	0.426	8.172	0.772	1.826	1.000	0.946	0.824
15	124.900^*^	<0.001	12.365	0.651	22.619	0.093	2.905	1.000	9.029	0.876
18	122.800^*^	<0.001	13.440	0.765	25.895	0.102	4.333	1.000	9.431	0.949
21	120.410^*^	<0.001	15.424	0.801	29.280	0.107	4.889	1.000	9.857	0.981
24	123.910^*^	<0.001	15.671	0.900	28.312	0.247	16.688	0.862	11.937	0.981
27	122.380^*^	<0.001	16.077	0.952	31.326	0.258	16.903	0.934	15.628	0.960
30	119.800^*^	<0.001	16.465	0.979	34.733	0.253	16.669	0.976	21.104	0.885
33	117.230^*^	<0.001	16.295	0.993	37.872	0.257	16.573	0.992	22.089	0.926
36	117.700^*^	<0.001	25.498	0.904	37.807	0.387	16.418	0.998	22.595	0.960

**Notes.**

* Signifies the LM-tests are statistically significant at the 5% level.

SARIMA seasonal autoregressive integrated moving average model NAR nonlinear auto-regressive neural network model NARX nonlinear auto-regressive with external input neural network

### The best-performing basic NAR model

**Figure 3:**
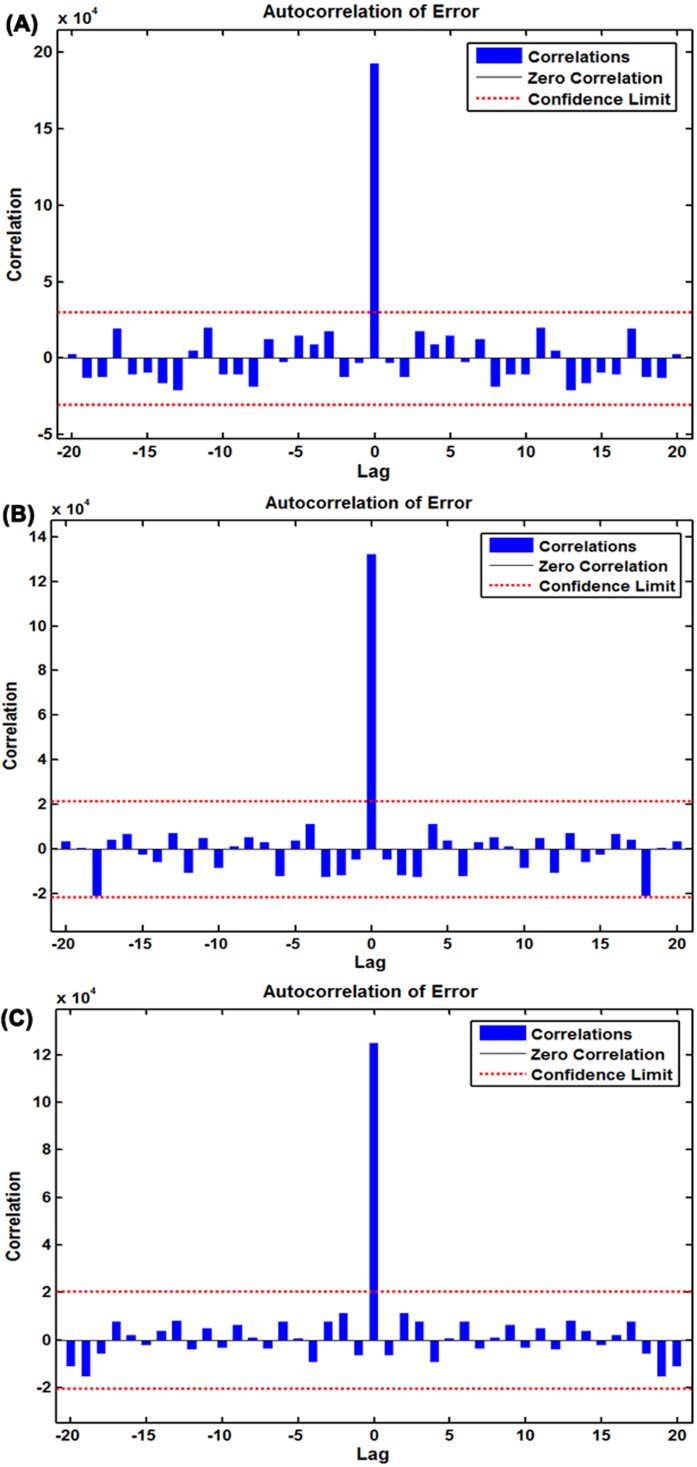
Autocorrelation function (ACF) plots of errors from various target series across varying lags. (A) ACF plot of errors from the basic NAR method; (B) ACF plot of errors from the SARIMA-NAR hybrid method; (C) ACF plot of errors from the SARIMA-NARX hybrid method. All of the correlations fell within the 95% uncertainty limits around zero across various lags except for the one at zero lag that should occur. Figures reveal the network may be suitable for the dataset.

**Figure 4:**
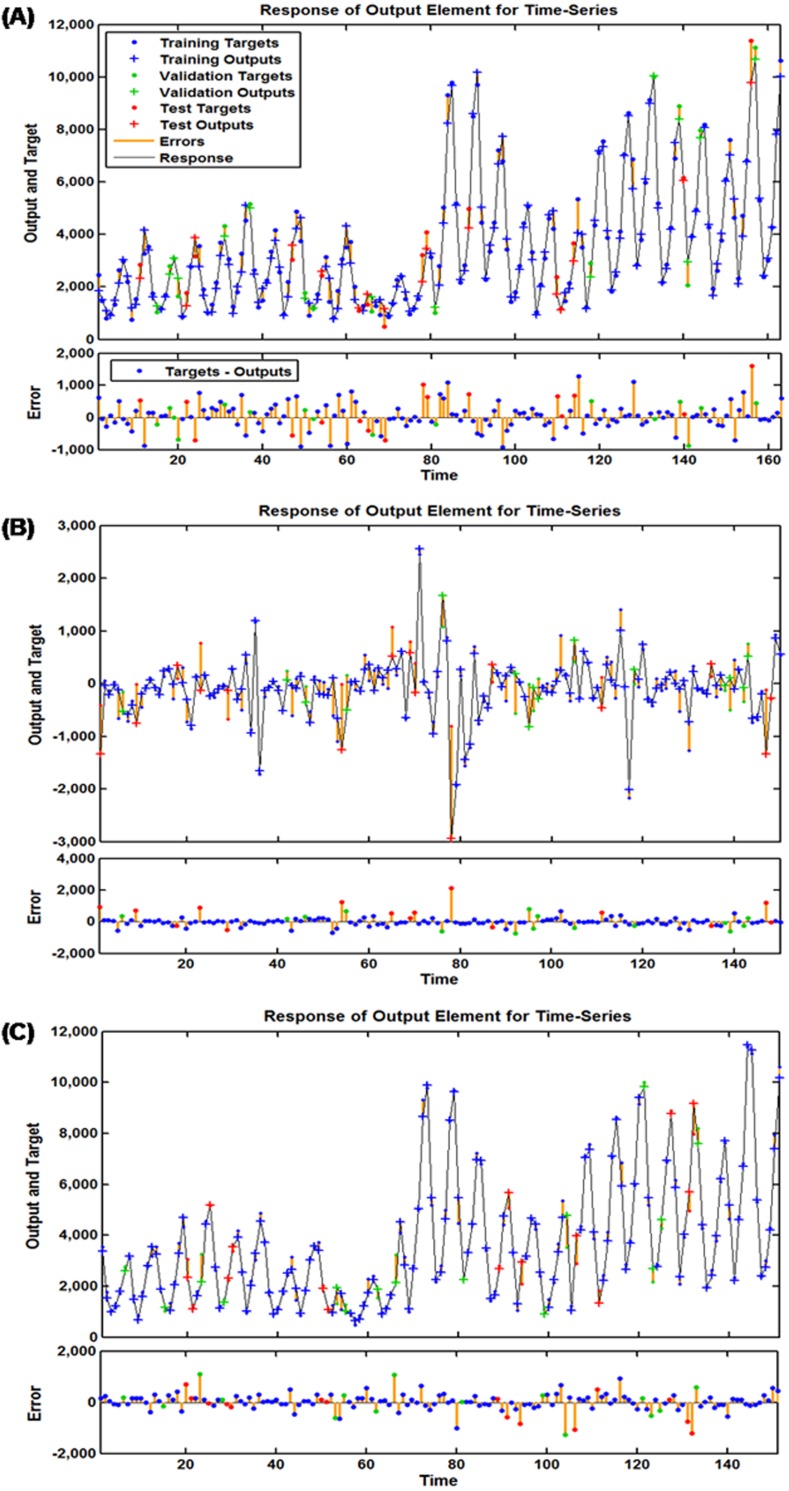
Response plots of inputs and targets at various time points for various target series. (A) Response plot for the basic NAR method; (B) response plot for the SARIMA-NAR hybrid method; (C) response plot for the SARIMA-NARX hybrid method. These graphs suggest which time points were utilized as the training, validation and testing subsets, along with their corresponding errors between inputs and targets. The small number of errors for the vast majority of points indicates that the selected network can be adopted to track future trends.

To discover a desired NAR technique, we repeatedly adjust the number of hidden units and feedback delays during training. After trying again and again, the architecture with 14 hidden neurons and five feedback delays should be taken into account the best-simulating basic NAR model according to the largest R values given by the training, validation, testing datasets and the entire dataset of 0.984, 0.993, 0.974, and 0.984, respectively (Fig. S7), together with the minimum MSE values of training for 160,229.489, validation for 174,582.498, testing for 472,659.037 and all data points for 192,306.305. To further test the suitability of the model, the results as presented in Fig. 3A and Table 1 demonstrated all autocorrelation coefficients remained individually dependent correlation at various lags aside from at zero lag where it should occur. The response graph of inputs and outputs manifested that the errors were acceptable in their corresponding subsets (Fig. 4A). Besides, the LM test also showed that the ARCH effect was removed from the residual errors series (Table 2). These aforementioned analyses provided further validation that this NAR model was applicable to the scarlet fever data.

### The optimal SARIMA-NAR combined model

Similar to the basic NAR approach, in an effort to train a series of networks by trial and error, the preferred ARIMA-NAR model with 33 hidden neurons and five feedback delays was found based on the lowest training score for MSE = 43,353.886, validation score for MSE = 220,899.525, testing score for MSE = 754,257.140 and entire dataset for MSE = 132,198.775, along with the maximum *R* values of training, validation, testing datasets and all data of 0.938, 0.620, 0.661, and 0.815, respectively (Fig. S8). Diagnostic checking for the erected model, the residual errors series was behaving like a white noise, visible in Fig. 3B, and the Ljung–Box *Q* test provided a further confirmation that the errors sequence met the need of a stochastic white noise (Table 1). The results given by the LM-test showed the volatility existed in the reported cases of scarlet fever could be wholly eliminated using this model (Table 2). The response plot of output elements for the randomly chosen training, validation and testing subsets suggested the overall epidemic pattern of scarlet fever morbidity was well captured by this method (Fig. 4B). In light of these diagnostic findings, this preferred method identified was worthy of being selected to forecast the future temporal trends of scarlet fever.

### The best-simulating SARIMA-NARX hybrid model

**Figure 5:**
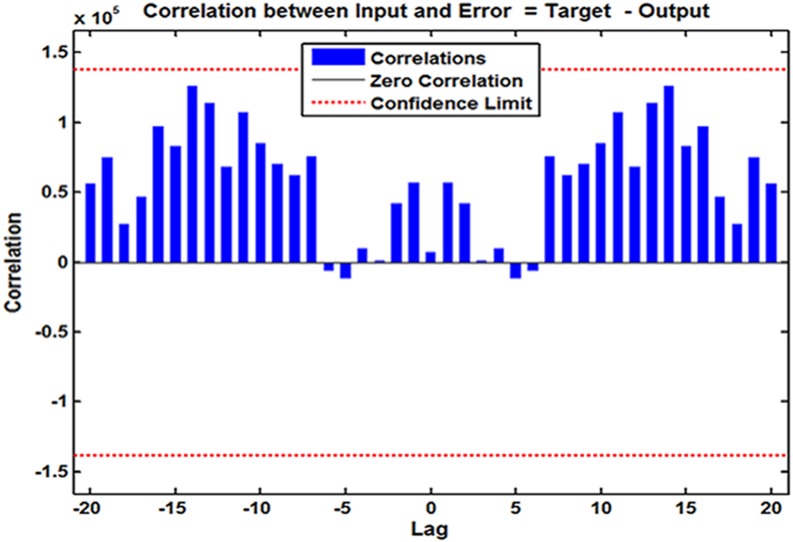
Input-to-error correlation plot across varying lags for SARIMA-NARX model. This input-error cross-correlation function indicates how the errors are correlated with the input series. For a perfect forecasting method, all of the correlations fall within the uncertainty limits around zero. The figure demonstrates that our developed model was perfect.

Following the modeling steps of this hybrid approach. After repeated attempts, such a SARIMA-NARX model with 16 hidden neurons and four feedback delays was identified as the preferred because this structure provided the optimal evaluation indicators of training score for MSE = 68,778.290, validation score for MSE = 360,821.711, testing score for MSE = 339,435.215, and all data for MSE = 124,675.675, coupled with the *R* values of training, validation, and testing datasets and all data of 0.997, 0.987, 0.940, and 0.992, respectively (Fig. S9). Further diagnostic analyses for the model: Looking at Fig. 3C, all spikes showed satisfactory results fallen within the 95% uncertainty limits and the *P* values from the Ljung–Box *Q* test were all greater than 0.05, meaning that the residuals successfully accomplished a white noise series (Table 1). As can be seen from Table 2, the ARCH effect was also not observed in the residual time series. The response graph is exhibited in Fig. 4C, demonstrating that the data were well fitted by this model because of the small errors. Furthermore, the input-error cross-correlation plot shows the inputs were not correlated with the errors, implying this was a perfect prediction (Fig. 5). The results obtained from the analyses above meant the elected configuration of the ARIMA-NARX was a perfect prediction model.

**Table 3:** Predicted morbidity numbers of scarlet fever from January 2018 to July 2018 with the selected four models.

**Time**	**Reportedcases**	**SARIMA**		**NAR**		**SARIMA-NAR**		**SARIMA-NARX**	
		**Forecastedcases**	**Relativeerror**	**Forecastedcases**	**Relativeerror**	**Forecastedcases**	**Relativeerror**	**Forecastedcases**	**Relativeerror**
January	7,564	7,039	0.069	7,351	0.028	7,418	0.019	6,312	0.166
February	2,159	2,998	0.389	2,955	0.369	2,534	0.173	2,190	0.014
March	3,774	5,816	0.541	4,355	0.154	5,603	0.485	4,088	0.083
April	6,784	8,759	0.291	5,129	0.244	7,810	0.151	5,602	0.174
May	10,747	13,938	0.297	7,358	0.315	14,288	0.329	12,516	0.165
June	10,716	14,393	0.343	6,885	0.358	14,855	0.386	13,388	0.249
July	5,385	7,584	0.408	3,671	0.318	7,393	0.373	5,475	0.017

**Notes.**

SARIMA seasonal autoregressive integrated moving average model NAR nonlinear auto-regressive neural network model NARX nonlinear auto-regressive with external input neural network

**Table 4:** Performance comparison among these four chosen models.

**Models**	**Simulated power**				**Predicted power**			
	**MAPE**	**RMSE**	**MER**	**RMSPE**	**MAPE**	**RMSE**	**MER**	**RMSPE**
SARIMA	0.152	609.323	0.110	1.1880	0.334	2,317.275	0.307	1.326
NAR	0.127	438.527	0.092	0.205	0.255	2,166.758	0.259	0.280
SARIMA-NAR	0.097	363.592	0.062	0.175	0.274	2,337.732	0.277	0.313
SARIMA-NARX	0.091	353.094	0.057	0.136	0.124	1,380.285	0.155	0.149

**Notes.**

SARIMA seasonal autoregressive integrated moving average model NAR nonlinear auto-regressive neural network model NARX nonlinear auto-regressive with external input neural network MAPE mean absolute percentage error RMSE root mean square error MER mean error rate RMSPE root mean square percentage error

**Figure 6:**
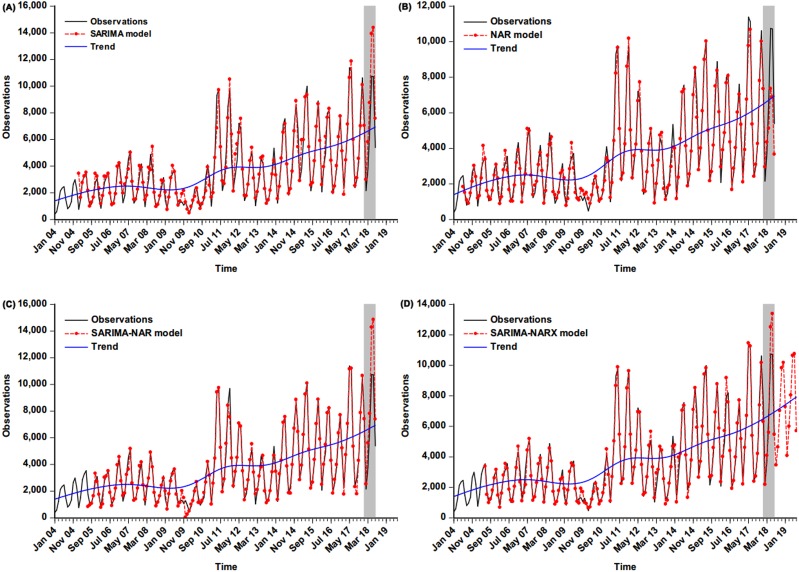
Comparison of incidence cases fitted and estimated between the selected four models and actual observations. (A) Comparison between the values from the basic SARIMA and the actual observations; (B) comparison between the values from the basic NAR and the actual observations; (C) comparison between the values from the SARIMA-NAR and the actual observations; (D) comparison between the values from the SARIMA-NARX and the actual observations. Overall, the figure suggest that the curve simulated and predicted by the SARIMA-NARX method (red line) was the closest to the actual observations (black line) among these four methods, and a continued rising trend was observed. The blue dotted line is the decomposed trend by the Hodrick-Prescott filter technique; the shaded area represents the validation dataset from January 2018 to July 2018; the red line outside of the shaded area in Fig. 6D represents the trends from August 2018 to July 2019 projected by the SARIMA-NARX method.

### Performance comparison among models

The four best-fitting methods developed were adopted to perform out-of-sample prediction, and subsequently by comparison with the performance of these models from two aspects of simulation and forecasting, the resulting results revealed that our proposed SARIMA-NARX hybrid model had the lowest values regarding MAPE, RMSE, MER and RMSPE (Tables 3 and 4). The ultimate fitting and predictive curves with the four selected methods are given in Fig. 6, Figs. S10 and S11, overall the curve from the SARIMA-NARX model was closer to the actual than the others as well. Based on the comparative analysis, the case numbers of scarlet fever from August 2018 to July 2019 were then estimated utilizing the best-presenting SARIMA-NARX technique (Figs. S12–S15, and Table S2).

## Discussion

In recent years, many countries have witnessed a growing scarlet fever case notifications, be it in the developed countries (Germany and England) or in the developing countries (China, Korea, Vietnam) (Andrey & Posfay-Barbe, 2016; Brockmann, Eichner & Eichner, 2018; Kim & Cheong, 2018; Lamagni et al., 2018; Liu et al., 2018). Therefore, in the current trend, the disease still remains a major public health issue. To tackle this, understanding the epidemic trajectories of this disease may play a significant role in the allocation of limited health resource and the formulation of prevention and control strategies. In this epidemiological research, we constructed four computational methods, a basic SARIMA, a basic NAR, a traditional SARIMA-NAR and a new SARIMA-NARX, and assessed their fitting and forecasting abilities utilizing the notified morbidity data of scarlet fever in mainland China. According to the mimic and forecasting accuracies, the SARIMA-NARX combined method mimics and predicts scarlet fever incidence better than the others. To our knowledge, no literature has proposed so far such a combined approach that integrated a SARIMA and NARX depended on the time factor, seen as an extension of the SARIMA-NAR, to identify the optimal method for predicting scarlet fever incidence; the desirable performance of the SARIMA-NARX combined method means the time driver can help to establish a greater degree of accuracy, and it should not be neglected in the forecasting process, which has provided a valuable insight into the domain of epidemiological prediction. As depicted before, the identification of key parameters for the four techniques plays a central role in the forecasting accuracy. In our current work, for the basic SARIMA method, it was considered both the ACF and PACF of the original observations and produced residuals to identify the preferred parameters (Fig. S16), as they can effectively capture the essence of the dependence between the current observations and the past observations, and the past observations under the condition of the given observation values, respectively, and thus providing important information regarding the scarlet fever notification series and its pattern formation. However, it should be noted that with the rapid development of computer simulation technology, many software components have currently provided a straightforward approach to automatically choose the optimal SARIMA model, like the “Expert Modeler” function in SPSS software, the “auto.arima()” function in R software and the “Auto-ARIMA forecasting” in EVIEWS and so forth. In contrast to the SARIMA method, for the basic NAR, traditional SARIMA-NAR and new SARIMA-NARX, there is a current lack of theoretical guidance to determine the number of hidden layer neurons, lagged periods and other parameters during the process of building ANNs models. If the number of hidden layer neurons is too small, the network cannot reflect the internal rule of time series. Otherwise the network training and learning time will be too long, and the generalization ability will be reduced. Therefore, in the practical application, there must be an optimal number of hidden layer neurons and lagged periods which need to be trained repeatedly to find the best-simulating network with them.

In our proposed combined approach, the linear SARIMA method and the nonlinear ANNs technique were jointly adopted, aimed at unearthing various types of the relationship in the disease series with distinct periodicity and seasonal variation so as to boost prediction capability. From this point of view, this SARIMA-NARX method can act as traction for early detecting and analyzing the temporal patterns, and can further facilitate the prevention and control of scarlet fever. Moreover, considering the desirable trait of low-cost data gathering of this model and its suitability for the application, we believe that it deserves to be extrapolated for forecasting other diseases displaying a strong seasonal variation and secular change. Nevertheless, with the rapid development of deep mining technology, numerous novel machine learning techniques have attracted much attention as a powerful modeling tool. For instance, a number of investigations to integrate modeling approaches like the back propagation neural network, generalized autoregressive model (GRNN), and long short-term memory network based on the discrete wavelet transform or ensemble empirical mode decomposition have showed an excellent potential to improve the performance in time series forecasting (Zhang et al., 2018a; Zhang et al., 2018b; Zhou et al., 2014b). Hence, further studies focusing on making a comparison between our proposed model and the above-mentioned methods need to be carried out in order to seek more precise forecasting techniques to explain the changing trends in the scarlet fever incidence. In addition, consistent with the past findings with reference to the predictions of tuberculosis (Wang et al., 2017), hand-foot-mouth disease (Yu et al., 2014) and schistosomiasis (Zhou et al., 2014a) using the SARIMA-NAR method, we found this combined technique has the capacity to outperform the basic NAR and SARIMA models in mimic stage. However, interestingly, in the forecasting stage, the method is only superior to the basic SARIMA model. The present finding is also supported by the earlier study which revealed that the SARIMA-NAR model is inferior to the basic NAR approach in the number of new admission inpatients forecasting (Zhou et al., 2018). Unfortunately, in contrast, the work involving the prediction of schistosomiasis prevalence failed to be in good agreement with the results of the present study (Zhou et al., 2014a). Likewise, the above findings were also observed in the most commonly used hybrid approach of the SARIMA-GRNN for the morbidity predictions of tuberculosis (Wei et al., 2017) and hemorrhagic fever with renal syndrome (Wu et al., 2015). These contradictory conclusions may be ascribed to the different characteristics of various infectious diseases from different areas, and also verify that the traditional SARIMA-NAR method is not always useful for estimating the morbidity of all infectious diseases, and it should be possible to improve the prediction of the traditional combined approaches under some circumstances. Therefore, it is necessary to develop a prediction model with high accuracy that is customized for different infectious diseases in various settings and at different time periods.

The results to emerge from this epidemiological study exhibited that a substantial rising trend in the scarlet fever case notifications was observed with an increase of 9.641% annually, particularly since 2011 with 12.869% annually, and there existed a marked seasonality in the scarlet fever case notifications from January 2004 to July 2018 in mainland China, with predominant peak activities of summer and winter. Among which seeing the lowest level of cases notified was in 2004 (1.457 per 100,000 population) and the highest level in 2017 (5.350 per 100,000 persons), the turning point with upsurge occurred in 2011 (4.741 per 100,000 population). During the period after sudden escalation, the reported cases have approximately increased by 2.279 times than that notified before sudden escalation. Albeit the current trend in the scarlet fever incidence is considerably upward the highest level is still much lower than other countries or regions or China’s previous epidemic periods (e.g., 33.2 per 100,000 population in England (Lamagni et al., 2018); 24.0 per 100,000 population in Hong Kong (Hsieh & Huang, 2011); 13.7 per 100,000 population in South Korea (Kim & Cheong, 2018); and 27.5 per 100,000 population in 1958 in China (You et al., 2018)). Under current trend, whether a skyrocketing trend will be continued in the near future still remains unclear. Consequently, the best-fitting SARIMA-NARX method was employed to perform short-term prediction for the incidence cases between August 2018 and July 2019. The method estimates a comparatively high morbidity cases, and moreover a mounting risk of persistent scarlet fever resurgence in the coming year in mainland China, meaning that a long-term countermeasure should be taken in advance as a reduction in the number of cases in the short term is unlikely. As for the striking rise, there appears to be several reasons: for one thing, it may stem from the fact that GAS antibiotic resistance and the change in circulating strains (Liu et al., 2018; You et al., 2018; Zhang et al., 2017), literature has suggested that despite the main group A streptococcus *emm* gene types are recognized to be different in some countries with ongoing resurgence of scarlet fever, the potential extension of a single clonal lineage or genetic elements within *S pyogenes* has been observed (Lamagni et al., 2018; Liu et al., 2018; Luk et al., 2012). In China, it has been reported that the *emm*12 gene possessed a high diversity of clones to which macrolides are highly resistant (You et al., 2018). More importantly, it was also found that the above-mentioned predominant genotypes and mobile elements were more dispersed geographically and annually than earlier deemed (You et al., 2018). Such diversity of *emm* genotypes leads to the lack of immunization against new circulating strains. For another thing, epidemic level of infectious diseases is often included a natural periodicity (Lamagni et al., 2018). Prior report has pointed out scarlet fever may be frequently followed by an epidemic periodicity of around every 6 years (You et al., 2018). While the epidemic behavior of scarlet fever retained a low morbidity in the period of 1995 through 2010 in China (Liu et al., 2018), hence the considerable increase observed in 2011 might be linked to this cyclic change. But it seems to have a longer cycle this time. Thirdly, with the two-child policy partially implemented in 2011 and officially implemented in 2016 in China, a booming growth in the susceptible subjects may be associated with such an escalation (Zeng & Hesketh, 2016). Fourthly, since 2004, the mandatory reporting requirements and gradual improvement in diagnostic tests for 39 statutory infectious diseases in mainland China may be partly responsible for this rise. Finally, scarlet fever fails still to be among vaccine preventable diseases until now. Besides, other possible mechanisms implicated in this appearance are subject to further investigation.

Seasonal patterns of contagious diseases are particularly valuable to infer temporal and spatiotemporal transmission parameters, which will help to better analyze and forecast the spread of the disease (Held & Paul, 2012). In the current research, a dual seasonal pattern was found in the scarlet fever data from mainland China, which peaked in May to June and November to December per year, and the first peak may be driven by a different epidemiological driver than the winter one. This may be related to different bacteria or changing risk (e.g., school attendance; weather). However, studies in relation to the difference are rare to find in literature. In the future what is needed are studies that should therefore concentrate on the investigation of this discrepancy. Our finding accords with earlier observations, and which further observed that the seasonal distribution of scarlet fever varied by the geographical location: The two peaks were primarily identified in the north and south of China, whereas the single peak in the southwest of China (Liu et al., 2018). The discrepancy of this seasonal distribution may due to the socioeconomic, environmental, and ecological factors. Similar seasonal pattern to our analysis was also reported in Hong Kong (Luk et al., 2012) and South Korea (Kim & Cheong, 2018). Yet differing in Poland and England, their peak activities occurred during the periods of January through March and January through March, respectively (Lamagni et al., 2018; Staszewska-Jakubik, Czarkowski & Kondej, 2016). In regard to the yearly incidence trough in February and July to October observed in the data, as reported in previous study (Liu et al., 2018), in all age groups, the infected individuals aged 3 to 6 years amount to the maximum proportion; thus, the summer and winter vacations, along with the Chinese New Year (the foremost festival annually in China that generally falls in mid-February) may be responsible for the low case notifications.

The advantage of the current study includes the longitudinal analysis with the scarlet fever incidence data covering 15 years based on the SARIMA-NARX method and provides a deep and reliable understanding of the trend and seasonal characteristics of scarlet fever. However, there are also several disadvantages: First, scarlet fever is currently recognized as mild illness, and seldom leads to death. The majority of mildly infected individuals are not accessible to healthcare professionals or are under diagnosed, thus resulting in under-reporting. Second, it is impossible to conduct further analysis owing to the lack of detailed information for scarlet fever notifications (e.g., age and sex). Third, other drivers associated with the occurrence and spread of scarlet fever are not included in our proposed model; hence, whether the model, which takes these variables into account, facilitates the improvement in the predictive accuracy will require further authentication. Fourth, the SARIMA-NARX approach is developed based on the benchmark model of SARIMA that is usually well suited to undertake short-term prediction. As such, to ensure that this combined technique provides the best estimation, the new reported data should be duly collected to update model. Finally, further researches may be warranted to demonstrate the potential of this approach and its suitability for the application in other infectious diseases.

## Conclusions

To conclude, our proposed SARIMA-NARX technique gets a more clear perspective of the scarlet fever incidence cases in both in-sample simulation and out-of-sample estimation than the traditional SARIMA-NAR, basic NAR and SARIMA methods. From the methodological facet, the model that we have identified can function as a profitable technology in predicting the incidence of scarlet fever, and therefore assist epidemiologists, health professionals and policymakers in providing early detection for epidemiological characteristics of scarlet fever in order to further optimize the allocation of resources relied on the advanced analysis for disease trends. Besides, given a growing risk of re-emerging scarlet fever in mainland China, specific strategies and countermeasures should be formulated to target this disease.

##  Supplemental Information

10.7717/peerj.6165/supp-1 File S1 Time series of monthly scarlet fever notified cases in mainland China from January 2004 to July 2018

10.7717/peerj.6165/supp-2 Figure S1 The architectural layout of the basic nonlinear autoregressive neural network (NAR) model (A) The opened loop form; (B) The closed loop form. This basic approach is made up of a hidden layer with 18 neurons and 5 delays and an output layer with 1 neuron. It is a two-layer feedforward network, with a sigmoid transfer function in the hidden layer and a linear transfer function in the output layer. The model has a single input that is applied to a tapped delay-line memory of d units. It has a single output that is fed back to the input via another tapped-delay-line memory, also of d units. The contents of these two tapped-delay-line memories are used to feed the input layer of the multilayer perceptron. The present value of the model input is denoted by *y*(*t* − 1), *y*(*t* − 2), …, *y*(*t* − *d*), and the corresponding value of the model output is denoted by y(t); that is, the output is ahead of the input by one time unit. Thus, the signal vector applied to the input layer of the multilayer perceptron consists of a data window made up of the components: the delayed values of the output, namely, *y*(*t* − 1), *y*(*t* − 2), …, *y*(*t* − *d*), on which the model output *y*(*t*) is regressed. Generally, in order to train more efficiently, the training can be undertaken in an open loop. Since the true output values are available during the course of training, we can use the open-loop architecture shown above (A), in which these values are employed instead of feeding back the projected outputs. This possesses two merits. The first is that the input to the feedforward network is more accurate. The second is that the resulting network has a purely feedforward architecture, and therefore a more efficient algorithm can be used for training. After training, then the opened loop form should be transformed to the closed loop form for multistep-ahead forecasting.

10.7717/peerj.6165/supp-3 Figure S2 The architectural l ayout of the ARIMA-NAR combined model (A) The opened loop form; (B) The closed loop form. This hybrid method is made up of a hidden layer with 33 neurons and 5 delays and an output layer with 1 neuron. It is a two-layer feedforward network, with a sigmoid transfer function in the hidden layer and a linear transfer function in the output layer. The model has a single input that is applied to a tapped delay-line memory of d units. It has a single output that is fed back to the input via another tapped-delay-line memory, also of d units. The contents of these two tapped-delay-line memories are used to feed the input layer of the multilayer perceptron. The present value of the model input is denoted by *e*(*t* − 1), *e*(*t* − 2), …, *e*(*t* − *d*), and the corresponding value of the model output is denoted by ¡!–[if !vml]–¿¡!–[endif]–¿; that is, the output is ahead of the input by one time unit. Thus, the signal vector applied to the input layer of the multilayer perceptron consists of a data window made up of the components: the delayed values of the output, namely, *e*(*t* − 1), *e*(*t* − 2), …, *e*(*t* − *d*), on which the model output ¡!–[if !vml]–¿¡!–[endif]–¿is regressed. Generally, in order to train more efficiently, the training can be undertaken in an open loop. Since the true output values are available in the process of the training of the technique, we can use the open-loop architecture shown above (a), in which these values are employed instead of feeding back the projected outputs. This has two advantages. The first is that the input to the feedforward network is more accurate. The second is that the resulting network has a purely feedforward architecture, and therefore a more efficient algorithm can be used for training. After training, then the opened loop form should be transformed to the closed loop form for multistep-ahead forecasting.

10.7717/peerj.6165/supp-4 Figure S3 The architectural layout of the novel ARIMA-NARX combined model (A) The opened loop form; (B) The closed loop form. This method is made up of a hidden layer with 16 neurons and 4 delays and an output layer with 1 neuron. It is a two-layer feedforward network with the default tan-sigmoid transfer function in the hidden layer and linear transfer function in the output layer. This network also uses tapped delay lines to store previous values of the *x*(*t*) and *y*(*t*) sequences. The contents of these tapped-delay-line memories are used to feed the input layer of the multilayer perceptron. The present value of the model input is denoted by *x*(*t*), and the corresponding value of the model output is denoted by *y*(*t*); that is, the output is ahead of the input by one time unit. Thus, the signal vector applied to the input layer of the multilayer perceptron consists of a data window made up of the components: the present and past values of the input, namely, *x*(*t* − 1), *x*(*t* − 2), …, *x*(*t* − *d*), which represent exogenous inputs originating from outside the network; the delayed values of the output, namely, *y*(*t* − 1), *y*(*t* − 2), …, *y*(*t* − *d*), on which the model output *y*(*t*) is regressed. Generally, for efficient training this feedback loop can be opened, the training can be undertaken in an open loop. Since the true output values are available in the process of the training of the technique, we can use the open-loop architecture shown above (a), in which these values are employed instead of feeding back the projected outputs. This has two advantages. The first is that the input to the feedforward network is more accurate. The second is that the resulting network has a purely feedforward architecture, and therefore a more efficient algorithm can be used for training. After training, then the opened loop form should be transformed to the closed loop form for multistep-ahead forecasting.

10.7717/peerj.6165/supp-5 Figure S4 The architectural layout of the novel ARIMA-NARX combined model (A) The opened loop form; (B) The closed loop form. This method is made up of a hidden layer with 16 neurons and 4 delays and an output layer with 1 neuron. It is a two-layer feedforward network with the default tan-sigmoid transfer function in the hidden layer and linear transfer function in the output layer. This network also uses tapped delay lines to store previous values of the *x*(*t*) and *y*(*t*) sequences. The contents of these tapped-d elay-line memories are used to feed the input layer of the multilayer perceptron. The present value of the model input is denoted by *x*(*t*), and the corresponding value of the model output is denoted by *y*(*t*); that is, the output is ahead of the input by one time unit. Thus, the signal vector applied to the input layer of the multilayer perceptron consists of a data window made up of the components: the present and past values of the input, namely, *x*(*t* − 1), *x*(*t* − 2), …, *x*(*t* − *d*), which represent exogenous inputs originating from outside the network; the delayed values of the output, namely, *y*(*t* − 1), *y*(*t* − 2), …, *y*(*t* − *d*), on which the model output *y*(*t*) is regressed. Generally, for efficient training this feedback loop can be opened, the training can be undertaken in an open loop. Since the true output values are available in the process of the training of the technique, we can use the open-loop architecture shown above (a), in which these values are employed instead of feeding back the projected outputs. This has two advantages. The first is that the input to the feedforward network is more accurate. The second is that the resulting network has a purely feedforward architecture, and therefore a more efficient algorithm can be used for training. After training, then the opened loop form should be transformed to the closed loop form for multistep-ahead forecasting.

10.7717/peerj.6165/supp-6 Figure S5 Trends from decomposing the monthly scarlet fever incidence time series using the Hodrick-Prescott filter method with monthly smoothing parameter *λ* = 14, 400 The red dotted line represents the decomposed trend by Hodrick-Prescott filter, a continued upside was observed after sudden upsurge, apart from cases notified in 2013.

10.7717/peerj.6165/supp-7 Figure S6 Seasonal factors from the monthly scarlet fever incidence time series from January 2004 to July 2018 using the additive seasonal decomposition technique Figure shows that there were few cases in February, a sharp increase in cases between March and June, high levels between May and June, with a decline in cases through July to October , but with a secondary peak during November and December of these years.

10.7717/peerj.6165/supp-8 Figure S7 The regression plots of the basic NAR model’s outputs corresponding to targets for the training, validation, testing and whole datasets

10.7717/peerj.6165/supp-9 Figure S8 The regression plots of the SARIMA-NAR model’s outputs corresponding to targets for the training, validation, testing and whole datasets

10.7717/peerj.6165/supp-10 Figure S9 The regression plots of the SARIMA-NARX model’s outputs corresponding to targets for the training, validation, testing and whole datasets

10.7717/peerj.6165/supp-11 Figure S10 Comparison of incidence cases fitted and estimated between the selected four models and actual observations Figure suggests overall the curve simulated and predicted by the SARIMA-NARX method (black line) was the closest to the actual observations (blue line) among these four methods, and a continued rising trend was observed. Among which the purple dotted line is the decomposed trend by the Hodrick-Prescott filter technique; The shaded area represents the validation dataset from January 2018 to July 2018 ; The black line outside of shaded area (right) represents the trends from August 2018 to July 2019 projected by the SARIMA-NARX method.

10.7717/peerj.6165/supp-12 Figure S11 The forecasted curves of the four models and the actual scarlet fever incidence series The blue line refers to the actual curve of scarlet fever, the black line is the curve projected by our proposed SARIMA-NARX combined method. Figure suggests that the curve forecasted by our proposed model is the closest to the actual.

10.7717/peerj.6165/supp-13 Figure S12 The regression plots of the SARIMA-NAR developed with all observations model’s outputs corresponding to targets for the training, validation, testing and whole datasets

10.7717/peerj.6165/supp-14 Figure S13 The response of inputs and targets for scarlet fever time series at various time points This graph suggests which time points were utilized as the training, validation and testing subsets, along with their corresponding errors between inputs and targets. Due to the small errors for vast majority of points indicating the selected network can be adopted to track future trends.

10.7717/peerj.6165/supp-15 Figure S14 Autocorrelation function (ACF) plot of errors across varying lags for SARIMA-NARX model All of the correlations fell within the 95% uncertainty limits around zero across various lags except for the one at zero lag that should occur. This showed the network may be suitable for the dataset.

10.7717/peerj.6165/supp-16 Figure S15 The input-to-error correlation plot across varying lags for SARIMA-NARX model This input-error cross-correlation function illustrates how the errors are correlated with the input sequence. For a perfect prediction model, all of the correlations fall within the confidence bounds around zero. Figure demonstrates that our developed model was perfect.

10.7717/peerj.6165/supp-17 Figure S16 Autocorrelation function(ACF) and partial autocorrelation function(PACF) plots across varying lags for the original incidence time series of scarlet fever (A) ACF plot: it describes the correlation between the time series observations and their past observations; (B) PACF plot : it describes the correlation between the time series observations and the past observations under the condition of given observation values . The autocorrelation coefficients can provide important information regarding the scarlet fever notification series and its pattern formation. For a random sequence, the autocorrelation coefficients of each order will be close to zero or equal to zero. The time series with obvious upward or downward trend or with strong seasonal or cyclic variation will have a strong autocorrelation. The usefulness of this information is that the autocorrelation coefficients of the existing time series data and their patterns can be obtained without any knowledge of the existing time series data, which can be used to reveal the characteristics of the time series data studied. And can help us to choose a suitable model. While, in the practical application, the ACF only provides a considerable amount of information about the order of the dependence when the process is a MA process. If the process , however, is AR, the ACF alone tells us little about the orders of dependence. At this time, the PACF should be used to judge the AR process. Hence, for an ARIMA or SARIMA model, it should be considered both the ACF and PACF to identify the preferred parameters. It can be seen from the graphs that the partial autocorrelations fall within the estimated 95% confidence limits at lag 23 in the PACF plot , whereas there still exist some local maximum values at lags 12, 24 and 36, and a reduced tendency for the autocorrelations out of the estimated 95% confidence limits at lag 40 in the ACF plot, being suggestive that of the reported scarlet fever notifications with strong seasonal and periodic characteristics. Therefore, it is necessary that the first-order seasonal and non-seasonal differences should be undertaken to obtain the stable mean and variance in this time series.

10.7717/peerj.6165/supp-18 Table S1 Time series of monthly scarlet fever notified cases in mainland China from January 2004 to July 2018

10.7717/peerj.6165/supp-19 Table S2 The LM and Ljung-Box Q tests of the residuals from the SARIMA-NARX model for the whole data at different lags

## Additional Information and Declarations

### Data Availability

The following information was supplied regarding data availability: Raw data is available in the Supplementary Files.
