# Supplementary figures and images for: Seasonality and trend prediction of scarlet fever incidence in mainland China from 2004 to 2018 using a hybrid SARIMA-NARX model
Source: PeerJ. 2019 Jan 17;7:e6165. doi: 10.7717/peerj.6165 (PMC6339779; doi:10.7717/peerj.6165)

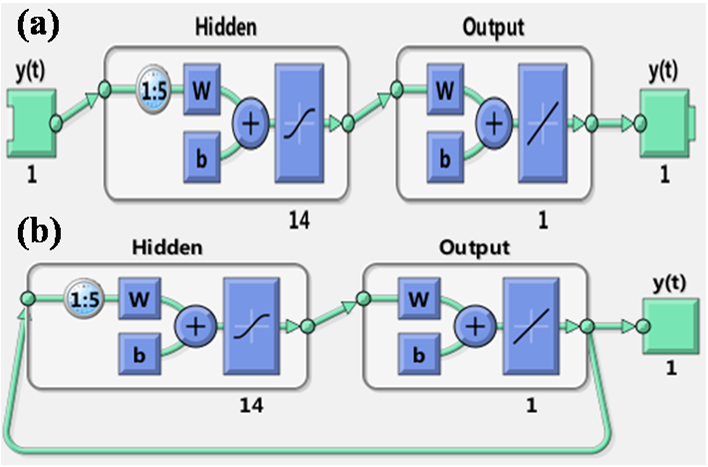

Supplement: Figure S1 — (A) The opened loop form; (B) The closed loop form. This basic approach is made up of a hidden layer with 18 neurons and 5 delays and an output layer with 1 neuron. It is a two-layer feedforward network, with a sigmoid transfer function in the hidden layer and a linear transfer function in the output layer. The model has a single input that is applied to a tapped delay-line memory of d units. It has a single output that is fed back to the input via another tapped-delay-line memory, also of d units. The contents of these two tapped-delay-line memories are used to feed the input layer of the multilayer perceptron. The present value of the model input is denoted by y(t − 1), y(t − 2), …, y(t − d), and the corresponding value of the model output is denoted by y(t); that is, the output is ahead of the input by one time unit. Thus, the signal vector applied to the input layer of the multilayer perceptron consists of a data window made up of the components: the delayed values of the output, namely, y(t − 1), y(t − 2), …, y(t − d), on which the model output y(t) is regressed. Generally, in order to train more efficiently, the training can be undertaken in an open loop. Since the true output values are available during the course of training, we can use the open-loop architecture shown above (A), in which these values are employed instead of feeding back the projected outputs. This possesses two merits. The first is that the input to the feedforward network is more accurate. The second is that the resulting network has a purely feedforward architecture, and therefore a more efficient algorithm can be used for training. After training, then the opened loop form should be transformed to the closed loop form for multistep-ahead forecasting. [file peerj-07-6165-s002.png]

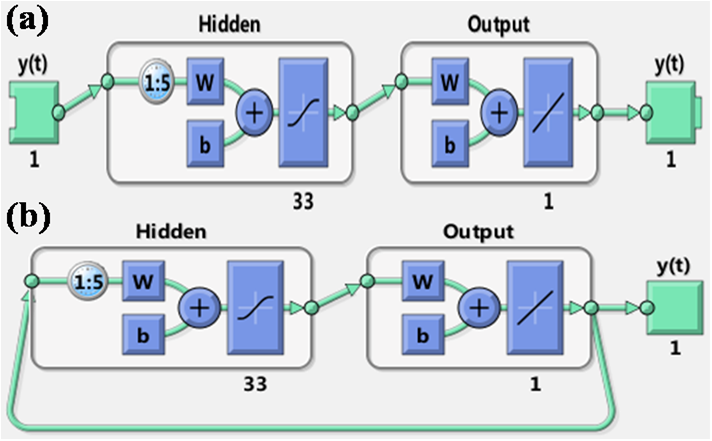

Supplement: Figure S2 — (A) The opened loop form; (B) The closed loop form. This hybrid method is made up of a hidden layer with 33 neurons and 5 delays and an output layer with 1 neuron. It is a two-layer feedforward network, with a sigmoid transfer function in the hidden layer and a linear transfer function in the output layer. The model has a single input that is applied to a tapped delay-line memory of d units. It has a single output that is fed back to the input via another tapped-delay-line memory, also of d units. The contents of these two tapped-delay-line memories are used to feed the input layer of the multilayer perceptron. The present value of the model input is denoted by e(t − 1), e(t − 2), …, e(t − d), and the corresponding value of the model output is denoted by ¡!–[if !vml]–¿¡!–[endif]–¿; that is, the output is ahead of the input by one time unit. Thus, the signal vector applied to the input layer of the multilayer perceptron consists of a data window made up of the components: the delayed values of the output, namely, e(t − 1), e(t − 2), …, e(t − d), on which the model output ¡!–[if !vml]–¿¡!–[endif]–¿is regressed. Generally, in order to train more efficiently, the training can be undertaken in an open loop. Since the true output values are available in the process of the training of the technique, we can use the open-loop architecture shown above (a), in which these values are employed instead of feeding back the projected outputs. This has two advantages. The first is that the input to the feedforward network is more accurate. The second is that the resulting network has a purely feedforward architecture, and therefore a more efficient algorithm can be used for training. After training, then the opened loop form should be transformed to the closed loop form for multistep-ahead forecasting. [file peerj-07-6165-s003.png]

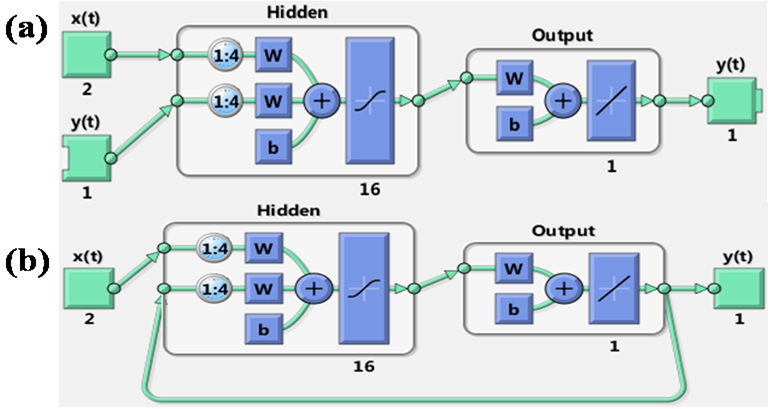

Supplement: Figure S3 — (A) The opened loop form; (B) The closed loop form. This method is made up of a hidden layer with 16 neurons and 4 delays and an output layer with 1 neuron. It is a two-layer feedforward network with the default tan-sigmoid transfer function in the hidden layer and linear transfer function in the output layer. This network also uses tapped delay lines to store previous values of the x(t) and y(t) sequences. The contents of these tapped-delay-line memories are used to feed the input layer of the multilayer perceptron. The present value of the model input is denoted by x(t), and the corresponding value of the model output is denoted by y(t); that is, the output is ahead of the input by one time unit. Thus, the signal vector applied to the input layer of the multilayer perceptron consists of a data window made up of the components: the present and past values of the input, namely, x(t − 1), x(t − 2), …, x(t − d), which represent exogenous inputs originating from outside the network; the delayed values of the output, namely, y(t − 1), y(t − 2), …, y(t − d), on which the model output y(t) is regressed. Generally, for efficient training this feedback loop can be opened, the training can be undertaken in an open loop. Since the true output values are available in the process of the training of the technique, we can use the open-loop architecture shown above (a), in which these values are employed instead of feeding back the projected outputs. This has two advantages. The first is that the input to the feedforward network is more accurate. The second is that the resulting network has a purely feedforward architecture, and therefore a more efficient algorithm can be used for training. After training, then the opened loop form should be transformed to the closed loop form for multistep-ahead forecasting. [file peerj-07-6165-s004.png]

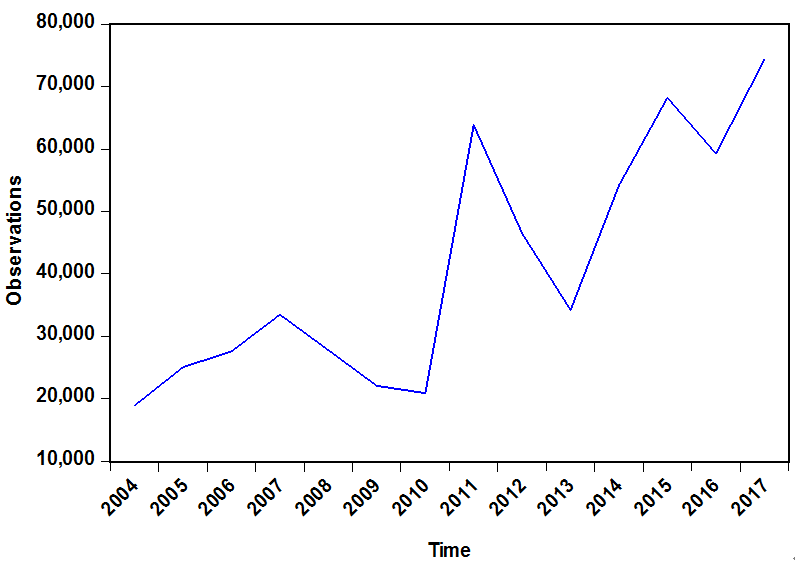

Supplement: Figure S4 — (A) The opened loop form; (B) The closed loop form. This method is made up of a hidden layer with 16 neurons and 4 delays and an output layer with 1 neuron. It is a two-layer feedforward network with the default tan-sigmoid transfer function in the hidden layer and linear transfer function in the output layer. This network also uses tapped delay lines to store previous values of the x(t) and y(t) sequences. The contents of these tapped-d elay-line memories are used to feed the input layer of the multilayer perceptron. The present value of the model input is denoted by x(t), and the corresponding value of the model output is denoted by y(t); that is, the output is ahead of the input by one time unit. Thus, the signal vector applied to the input layer of the multilayer perceptron consists of a data window made up of the components: the present and past values of the input, namely, x(t − 1), x(t − 2), …, x(t − d), which represent exogenous inputs originating from outside the network; the delayed values of the output, namely, y(t − 1), y(t − 2), …, y(t − d), on which the model output y(t) is regressed. Generally, for efficient training this feedback loop can be opened, the training can be undertaken in an open loop. Since the true output values are available in the process of the training of the technique, we can use the open-loop architecture shown above (a), in which these values are employed instead of feeding back the projected outputs. This has two advantages. The first is that the input to the feedforward network is more accurate. The second is that the resulting network has a purely feedforward architecture, and therefore a more efficient algorithm can be used for training. After training, then the opened loop form should be transformed to the closed loop form for multistep-ahead forecasting. [file peerj-07-6165-s005.png]

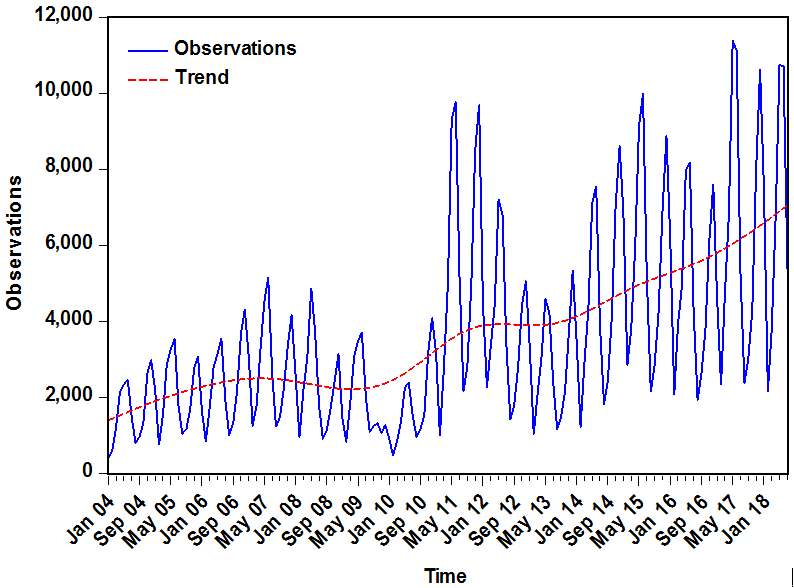

Supplement: Figure S5 — The red dotted line represents the decomposed trend by Hodrick-Prescott filter, a continued upside was observed after sudden upsurge, apart from cases notified in 2013. [file peerj-07-6165-s006.png]

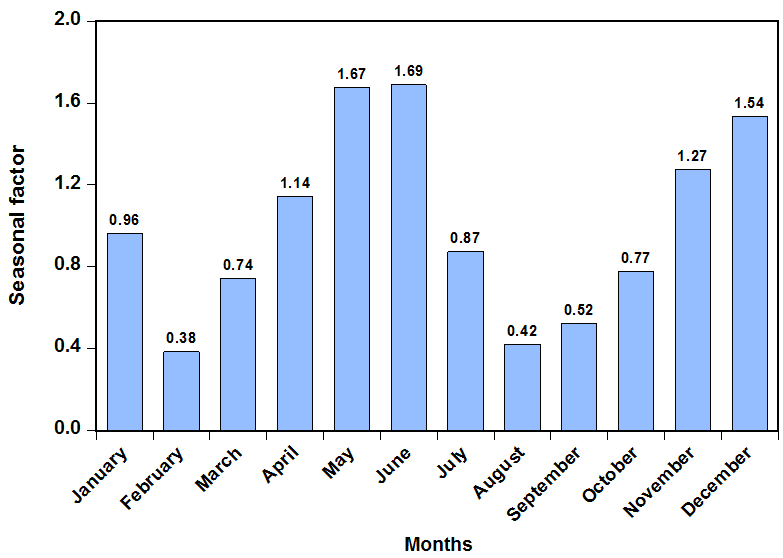

Supplement: Figure S6 — Figure shows that there were few cases in February, a sharp increase in cases between March and June, high levels between May and June, with a decline in cases through July to October , but with a secondary peak during November and December of these years. [file peerj-07-6165-s007.png]

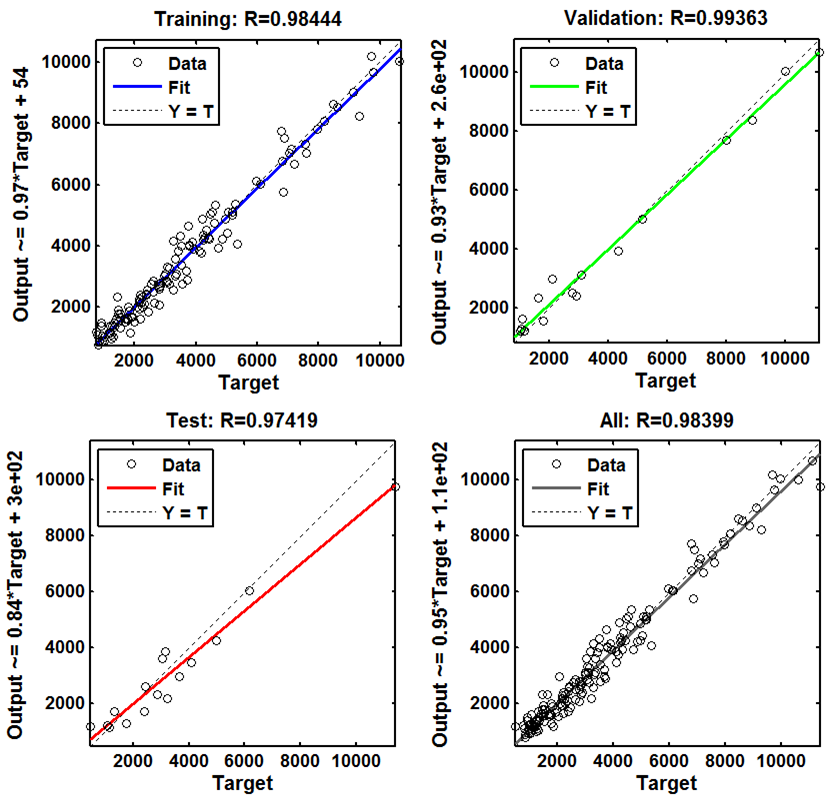

Supplement: Figure S7 [file peerj-07-6165-s008.png]

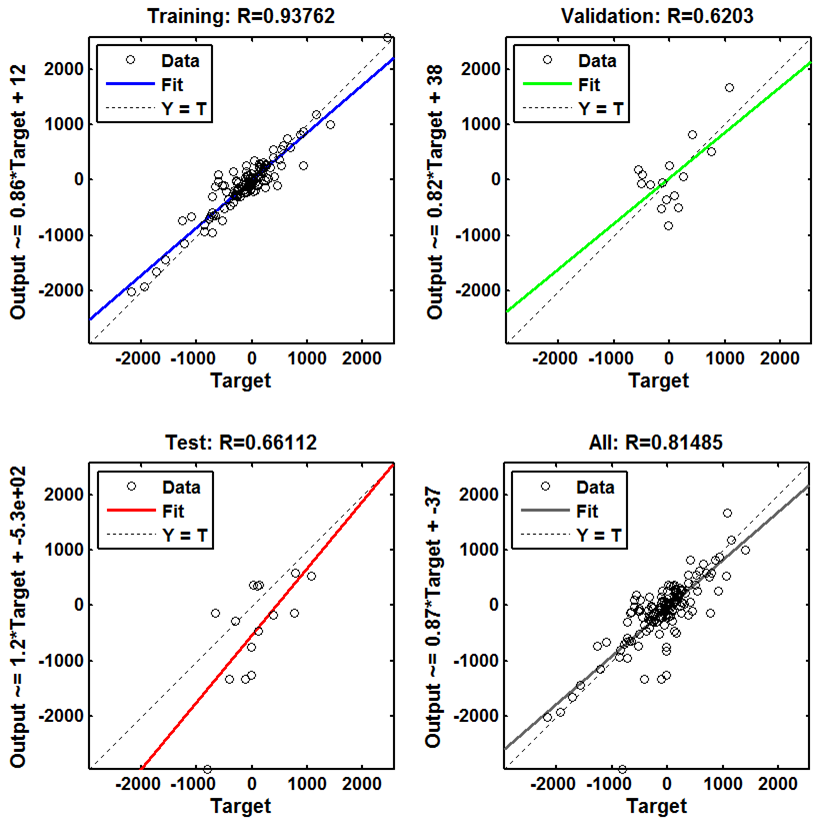

Supplement: Figure S8 [file peerj-07-6165-s009.png]

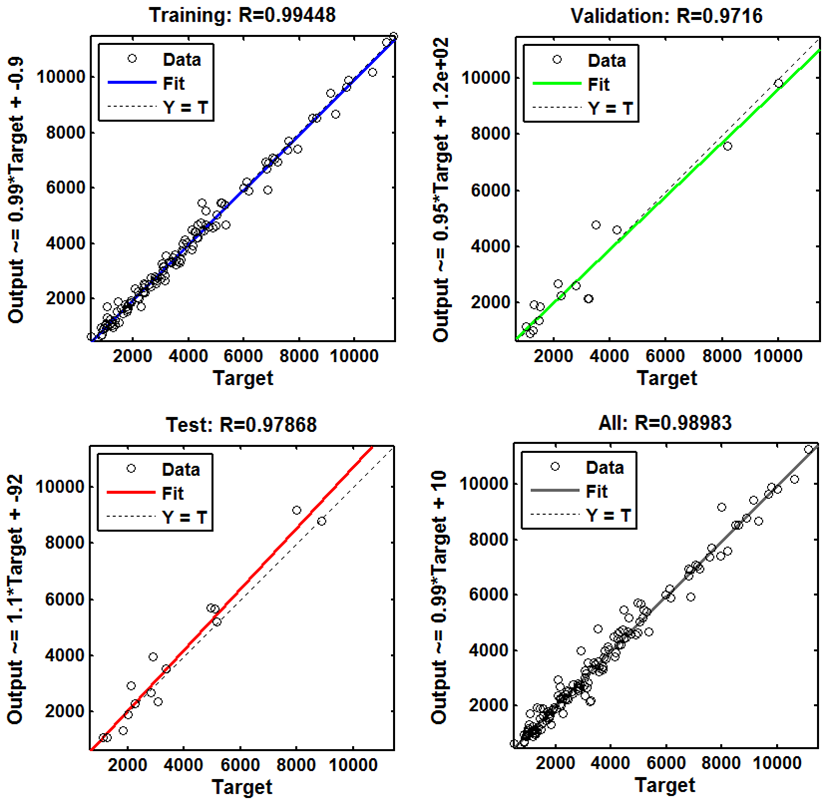

Supplement: Figure S9 [file peerj-07-6165-s010.png]

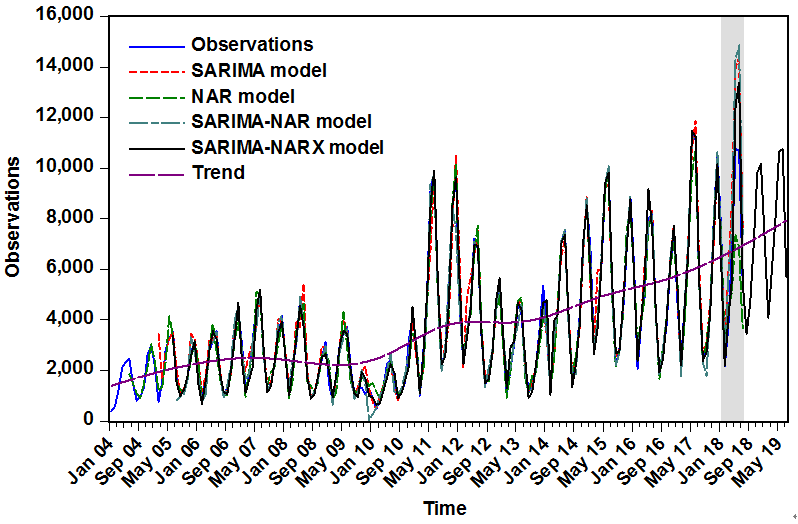

Supplement: Figure S10 — Figure suggests overall the curve simulated and predicted by the SARIMA-NARX method (black line) was the closest to the actual observations (blue line) among these four methods, and a continued rising trend was observed. Among which the purple dotted line is the decomposed trend by the Hodrick-Prescott filter technique; The shaded area represents the validation dataset from January 2018 to July 2018 ; The black line outside of shaded area (right) represents the trends from August 2018 to July 2019 projected by the SARIMA-NARX method. [file peerj-07-6165-s011.png]

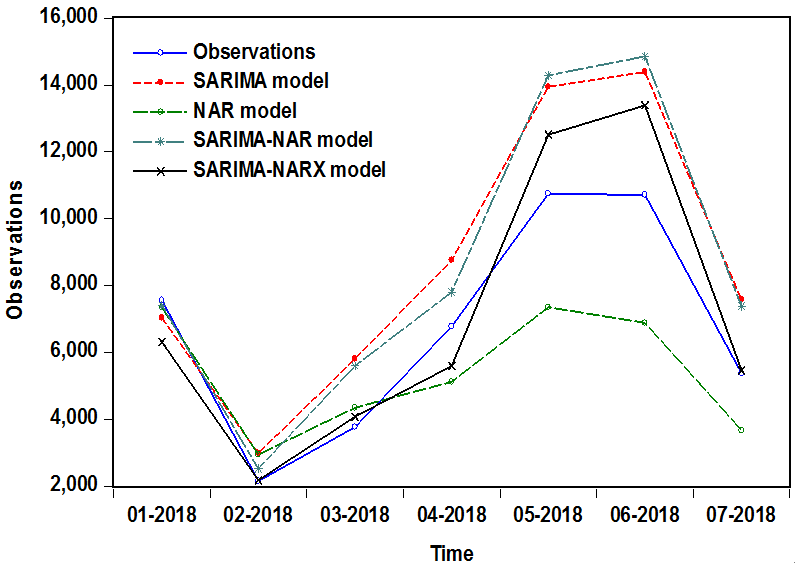

Supplement: Figure S11 — The blue line refers to the actual curve of scarlet fever, the black line is the curve projected by our proposed SARIMA-NARX combined method. Figure suggests that the curve forecasted by our proposed model is the closest to the actual. [file peerj-07-6165-s012.png]

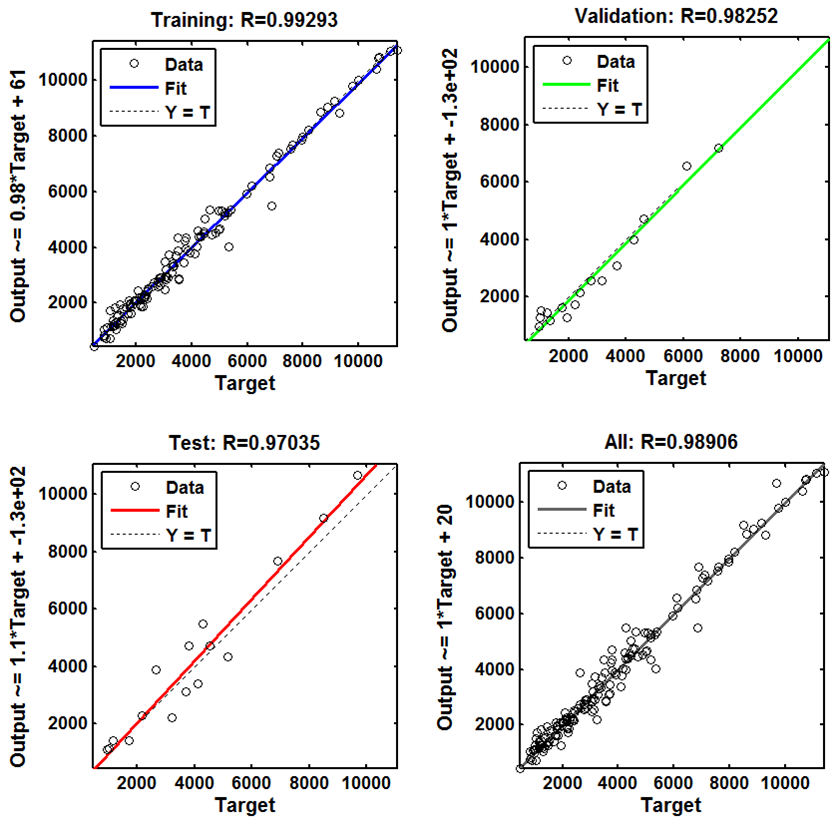

Supplement: Figure S12 [file peerj-07-6165-s013.png]

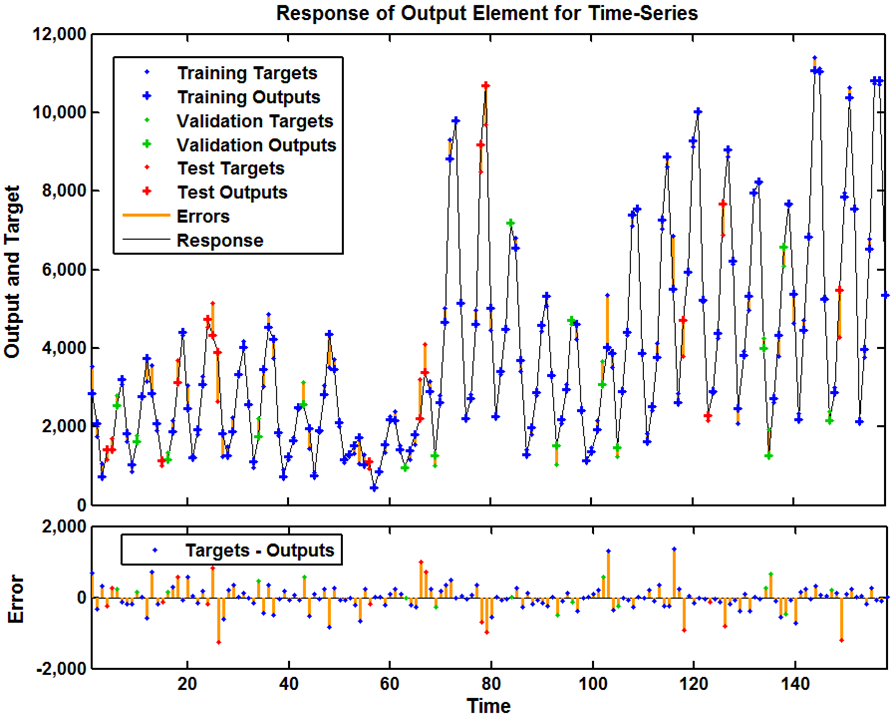

Supplement: Figure S13 — This graph suggests which time points were utilized as the training, validation and testing subsets, along with their corresponding errors between inputs and targets. Due to the small errors for vast majority of points indicating the selected network can be adopted to track future trends. [file peerj-07-6165-s014.png]

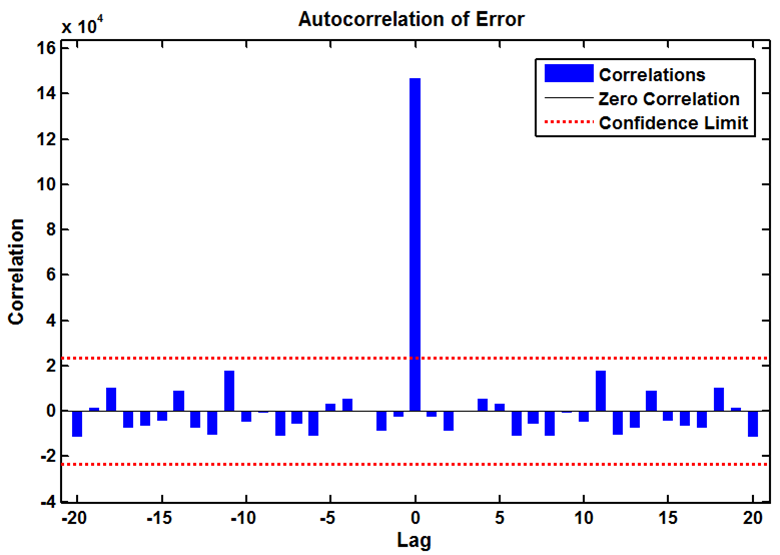

Supplement: Figure S14 — All of the correlations fell within the 95% uncertainty limits around zero across various lags except for the one at zero lag that should occur. This showed the network may be suitable for the dataset. [file peerj-07-6165-s015.png]

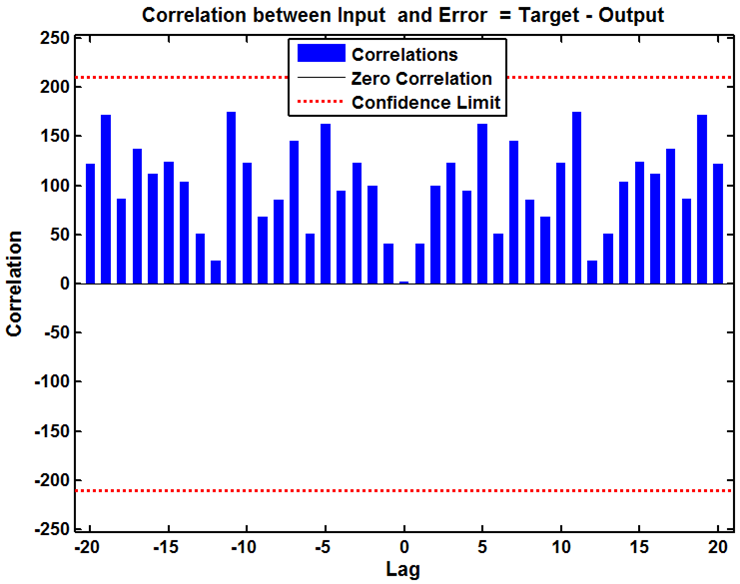

Supplement: Figure S15 — This input-error cross-correlation function illustrates how the errors are correlated with the input sequence. For a perfect prediction model, all of the correlations fall within the confidence bounds around zero. Figure demonstrates that our developed model was perfect. [file peerj-07-6165-s016.png]

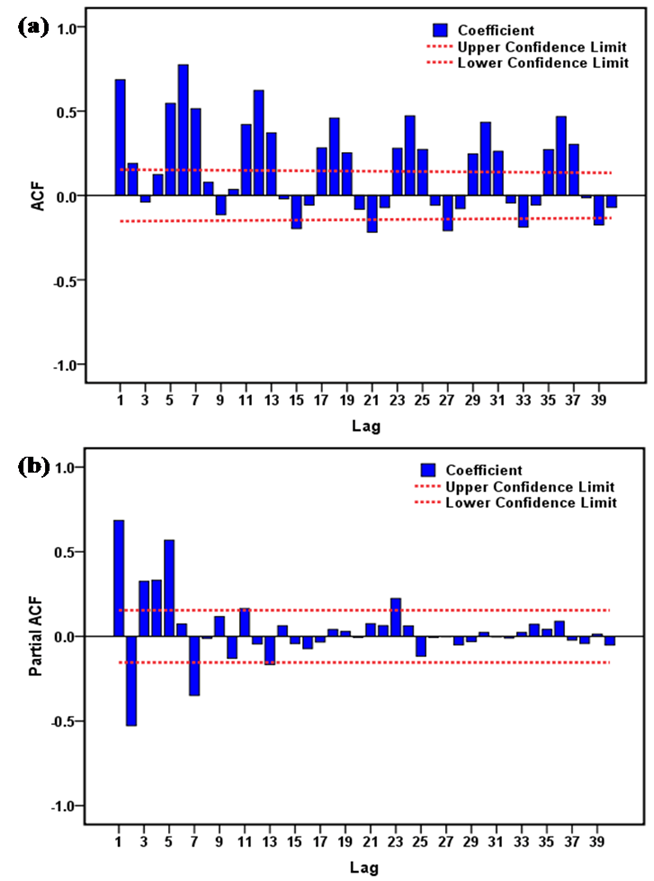

Supplement: Figure S16 — (A) ACF plot: it describes the correlation between the time series observations and their past observations; (B) PACF plot : it describes the correlation between the time series observations and the past observations under the condition of given observation values . The autocorrelation coefficients can provide important information regarding the scarlet fever notification series and its pattern formation. For a random sequence, the autocorrelation coefficients of each order will be close to zero or equal to zero. The time series with obvious upward or downward trend or with strong seasonal or cyclic variation will have a strong autocorrelation. The usefulness of this information is that the autocorrelation coefficients of the existing time series data and their patterns can be obtained without any knowledge of the existing time series data, which can be used to reveal the characteristics of the time series data studied. And can help us to choose a suitable model. While, in the practical application, the ACF only provides a considerable amount of information about the order of the dependence when the process is a MA process. If the process , however, is AR, the ACF alone tells us little about the orders of dependence. At this time, the PACF should be used to judge the AR process. Hence, for an ARIMA or SARIMA model, it should be considered both the ACF and PACF to identify the preferred parameters. It can be seen from the graphs that the partial autocorrelations fall within the estimated 95% confidence limits at lag 23 in the PACF plot , whereas there still exist some local maximum values at lags 12, 24 and 36, and a reduced tendency for the autocorrelations out of the estimated 95% confidence limits at lag 40 in the ACF plot, being suggestive that of the reported scarlet fever notifications with strong seasonal and periodic characteristics. Therefore, it is necessary that the first-order seasonal and non-seasonal differences should be undertaken to obtain the stable [file peerj-07-6165-s017.png]
