# Supplementary material for: Seasonality and trend prediction of scarlet fever incidence in mainland China from 2004 to 2018 using a hybrid SARIMA-NARX model
Source: PeerJ. 2019 Jan 17;7:e6165. doi: 10.7717/peerj.6165 (PMC6339779; doi:10.7717/peerj.6165)
Supplement: Table S1 [file peerj-07-6165-s018.docx]

| Data | Cases | Data | Cases | Data | Cases | Data | Cases | Data | Cases |
| --- | --- | --- | --- | --- | --- | --- | --- | --- | --- |
| 01-2004 | 387 | 12-2006 | 4326 | 11-2009 | 1065 | 10-2012 | 2804 | 09-2015 | 2854 |
| 02-2004 | 588 | 01-2007 | 3052 | 12-2009 | 1283 | 11-2012 | 4420 | 10-2015 | 4256 |
| 03-2004 | 1229 | 02-2007 | 1255 | 01-2010 | 925 | 12-2012 | 5066 | 11-2015 | 6883 |
| 04-2004 | 2131 | 03-2007 | 1792 | 02-2010 | 473 | 01-2013 | 3316 | 12-2015 | 8878 |
| 05-2004 | 2352 | 04-2007 | 3269 | 03-2010 | 853 | 02-2013 | 1037 | 01-2016 | 6152 |
| 06-2004 | 2464 | 05-2007 | 4538 | 04-2010 | 1352 | 03-2013 | 2090 | 02-2016 | 2074 |
| 07-2004 | 1469 | 06-2007 | 5153 | 05-2010 | 2269 | 04-2013 | 3073 | 03-2016 | 3931 |
| 08-2004 | 805 | 07-2007 | 2632 | 06-2010 | 2397 | 05-2013 | 4600 | 04-2016 | 4955 |
| 09-2004 | 964 | 08-2007 | 1232 | 07-2010 | 1526 | 06-2013 | 4226 | 05-2016 | 7980 |
| 10-2004 | 1339 | 09-2007 | 1482 | 08-2010 | 958 | 07-2013 | 2389 | 06-2016 | 8190 |
| 11-2004 | 2643 | 10-2007 | 2239 | 09-2010 | 1169 | 08-2013 | 1168 | 07-2016 | 4256 |
| 12-2004 | 2989 | 11-2007 | 3360 | 10-2010 | 1541 | 09-2013 | 1465 | 08-2016 | 1930 |
| 01-2005 | 2205 | 12-2007 | 4166 | 11-2010 | 3217 | 10-2013 | 2150 | 09-2016 | 2624 |
| 02-2005 | 761 | 01-2008 | 2563 | 12-2010 | 4094 | 11-2013 | 3659 | 10-2016 | 3781 |
| 03-2005 | 1545 | 02-2008 | 961 | 01-2011 | 3143 | 12-2013 | 5346 | 11-2016 | 6097 |
| 04-2005 | 2853 | 03-2008 | 2198 | 02-2011 | 1000 | 01-2014 | 3513 | 12-2016 | 7611 |
| 05-2005 | 3274 | 04-2008 | 3036 | 03-2011 | 2795 | 02-2014 | 1230 | 01-2017 | 4633 |
| 06-2005 | 3540 | 05-2008 | 4874 | 04-2011 | 5023 | 03-2014 | 2890 | 02-2017 | 2335 |
| 07-2005 | 1757 | 06-2008 | 3740 | 05-2011 | 9308 | 04-2014 | 4338 | 03-2017 | 4718 |
| 08-2005 | 1048 | 07-2008 | 1783 | 06-2011 | 9773 | 05-2014 | 7113 | 04-2017 | 6804 |
| 09-2005 | 1165 | 08-2008 | 914 | 07-2011 | 5173 | 06-2014 | 7557 | 05-2017 | 11388 |
| 10-2005 | 1694 | 09-2008 | 1156 | 08-2011 | 2174 | 07-2014 | 3873 | 06-2017 | 11129 |
| 11-2005 | 2784 | 10-2008 | 1713 | 09-2011 | 2813 | 08-2014 | 1827 | 07-2017 | 5298 |
| 12-2005 | 3087 | 11-2008 | 2431 | 10-2011 | 4972 | 09-2014 | 2424 | 08-2017 | 2379 |
| 01-2006 | 1629 | 12-2008 | 3138 | 11-2011 | 8488 | 10-2014 | 4118 | 09-2017 | 3006 |
| 02-2006 | 848 | 01-2009 | 1440 | 12-2011 | 9696 | 11-2014 | 7043 | 10-2017 | 4279 |
| 03-2006 | 1768 | 02-2009 | 834 | 01-2012 | 4460 | 12-2014 | 8615 | 11-2017 | 7947 |
| 04-2006 | 2783 | 03-2009 | 1859 | 02-2012 | 2268 | 01-2015 | 6859 | 12-2017 | 10622 |
| 05-2006 | 3163 | 04-2009 | 3059 | 03-2012 | 3339 | 02-2015 | 2858 | 01-2018 | 7564 |
| 06-2006 | 3550 | 05-2009 | 3500 | 04-2012 | 4443 | 03-2015 | 3788 | 02-2018 | 2159 |
| 07-2006 | 1911 | 06-2009 | 3712 | 05-2012 | 7210 | 04-2015 | 5968 | 03-2018 | 3774 |
| 08-2006 | 1009 | 07-2009 | 2013 | 06-2012 | 6794 | 05-2015 | 9130 | 04-2018 | 6784 |
| 09-2006 | 1328 | 08-2009 | 1096 | 07-2012 | 3416 | 06-2015 | 9992 | 05-2018 | 10747 |
| 10-2006 | 2165 | 09-2009 | 1262 | 08-2012 | 1418 | 07-2015 | 5176 | 06-2018 | 10716 |
| 11-2006 | 3692 | 10-2009 | 1318 | 09-2012 | 1792 | 08-2015 | 2156 | 07-2018 | 5385 |
