# Supplementary material for: Seasonality and trend prediction of scarlet fever incidence in mainland China from 2004 to 2018 using a hybrid SARIMA-NARX model
Source: PeerJ. 2019 Jan 17;7:e6165. doi: 10.7717/peerj.6165 (PMC6339779; doi:10.7717/peerj.6165)
Supplement: Table S2 [file peerj-07-6165-s019.docx]

| **Lags** | **Observed values** | | | |  | **Residuals of SARIMA-NARX model** | | | |
| --- | --- | --- | --- | --- | --- | --- | --- | --- | --- |
|  | **Box-Ljung Q** | ***P*** | **LM-test** | ***P*** |  | **Box-Ljung Q** | ***P*** | **LM-test** | ***P*** |
| 1 | 87.766^*^ | <0.001 | 70.650^*^ | <0.001 |  | 0.049 | 0.824 | 2.121 | 0.145 |
| 3 | 95.415^*^ | <0.001 | 106.390^*^ | <0.001 |  | 0.661 | 0.882 | 4.766 | 4.766 |
| 6 | 265.243^*^ | <0.001 | 135.230^*^ | <0.001 |  | 1.795 | 0.938 | 8.011 | 0.237 |
| 9 | 318.791^*^ | <0.001 | 133.750^*^ | <0.001 |  | 2.943 | 0.967 | 11.773 | 0.226 |
| 12 | 441.271^*^ | <0.001 | 135.470^*^ | <0.001 |  | 6.033 | 0.914 | 12.888 | 0.377 |
| 15 | 481.569^*^ | <0.001 | 134.720^*^ | <0.001 |  | 7.063 | 0.956 | 21.576 | 0.119 |
| 18 | 538.302^*^ | <0.001 | 132.580^*^ | <0.001 |  | 8.409 | 0.972 | 22.043 | 0.230 |
| 21 | 560.983^*^ | <0.001 | 130.140^*^ | <0.001 |  | 9.423 | 0.985 | 25.564 | 0.224 |
| 24 | 627.764^*^ | <0.001 | 132.820^*^ | <0.001 |  | 16.766 | 0.858 | 27.226 | 0.294 |
| 27 | 653.602^*^ | <0.001 | 131.180^*^ | <0.001 |  | 16.803 | 0.936 | 31.425 | 0.254 |
| 30 | 713.508^*^ | <0.001 | 128.530^*^ | <0.001 |  | 17.248 | 0.969 | 33.063 | 0.320 |
| 33 | 737.235^*^ | <0.001 | 125.880^*^ | <0.001 |  | 21.257 | 0.943 | 36.247 | 0.320 |
| 36 | 816.771^*^ | <0.001 | 125.850^*^ | <0.001 |  | 21.540 | 0.973 | 39.305 | 0.324 |

Note:* denotes the Box-Ljung Q and LM tests are statistically significant at the 5% level
